# Dangguibuxue Decoction Attenuated AA I-Induced Renal Fibrosis: Integrating Network Pharmacology and Experimental Validation

**DOI:** 10.3390/ph19081206

**Published:** 2026-08-01

**Authors:** Suyan Liu, Jing Meng, Yong Zhao, Chunying Li, Yan Yi, Jiayin Han, Yushi Zhang, Chen Pan, Xingwen Wang, Liping Wang, Feng Gao, Xingnan Yue, Jingwen Wu, Hongmei Li, Aihua Liang

**Affiliations:** 1 State Key Laboratory for Quality Ensurance and Sustainable Use of Dao-di Herbs, Institute of Chinese Materia Medica, China Academy of Chinese Medical Sciences, Beijing 100700, China; syliu@icmm.ac.cn (S.L.); jyhan@icmm.ac.cn (J.H.); 2 Beijing Key Laboratory of Psychoactive Substances Discovery and Control in Chinese Herbal Medicines, Beijing 100700, China; icmmmj@163.com (J.M.); yzhao@icmm.ac.cn (Y.Z.); cyli@icmm.ac.cn (C.L.); yyi@icmm.ac.cn (Y.Y.); yszhang@icmm.ac.cn (Y.Z.); cpan@icmm.ac.cn (C.P.); xwwang@icmm.ac.cn (X.W.); 13107626750@163.com (L.W.); fgao@icmm.ac.cn (F.G.); yuexingnan1226@163.com (X.Y.); nnniwu333@gmail.com (J.W.); 3 Key Laboratory of TCM Safety Risk Assessment and Translation, China Academy of Chinese Medical Sciences, Beijing 100700, China

**Keywords:** Dangguibuxue decoction, renal fibrosis, hypoxia, TGF-β/Smad, phosphorylation, rutin

## Abstract

**Background**: Dangguibuxue decoction (DD), containing *Angelica sinensis* (Oliv.) Diels (AS) and *Astragalus membranaceus* (Fisch.) Bge. (AM) (1:5), is a well-known traditional Chinese medicine (TCM) used for strengthening qi and nourishing the blood. DD has shown therapeutic effects in nephropathy patients. However, the underlying mechanisms based on the traditional efficacy are still not fully elucidated. **Methods**: The chemical constituents in DD were identified using UPLC-MS/MS. Network pharmacology analysis was applied to predict the potential target genes and associated signaling pathways. A renal fibrosis mouse model was induced by the intraperitoneal injection of aristolochic acid I (AA I) at 3.0 mg/kg. Mice were treated with AM, AS, and DD at two dosages by oral gavage for 30 days. Body weights, serum biochemistry, hematology, and histopathology observations were assessed. The key targets predicted were validated using qRT-PCR and Western blotting. The active constituents were screened by molecular docking, and their anti-fibrotic effects were evaluated through in vitro assays. **Results**: DD effectively improved renal functions and alleviated AA I-induced renal fibrosis. DD alleviated anemia and upregulated the expression of Erythropoietin (EPO). Network pharmacology analysis indicated the involvement of signaling pathways, including the PI3K/Akt, hypoxia-inducible factor-1α (HIF-1α) and transforming growth factor-β (TGF-β) signaling pathways. Experimental validation further demonstrated that DD reduced the protein expression of HIF-1α, collagen I, and TGF-β, and the ratios of phosphorylated Smad2/3 to total Smad2/3. Molecular docking and in vitro assays suggested that rutin may be a potential bioactive compound in DD. **Conclusions**: This research indicated that DD ameliorated AA I-induced renal fibrosis in mice, which may be associated with the modulation of HIF-1α and TGF-β/Smad signaling pathways. Rutin may be a potential bioactive compound in DD with anti-fibrotic activity, but further studies are still needed to clarify the content of rutin in DD, the amount of its exposure in the body, and its contribution to the effects of DD.

## 1. Introduction

Chronic kidney disease (CKD), known as the “silent killer” for its lack of well-defined symptoms, is gradually becoming a worldwide health burden [1,2]. Renal fibrosis, characterized by myofibroblast activation, epithelial-to-mesenchymal transition (EMT), excessive production and accumulation of the extracellular matrix (ECM), and loss of functioning nephrons, is a common pathological characteristic of CKD as it progresses to end-stage renal failure (ESRD). Renal fibrosis processes dynamically, under the influence of complicated factors and multiple mechanisms and pathways [3]. Among these, transforming growth factor-β (TGF-β) is regarded as a crucial regulator in the progression of renal fibrosis. TGF-β regulates renal fibrosis through canonical (Smad-dependent) and noncanonical (Smad-independent) signaling pathways by activating myofibroblasts and inducing excessive production of ECM, while inhibiting ECM degradation [4]. In the downstream signaling pathway of TGF-β, the Smad signaling pathway is regarded as the most crucial pathway, with Smad 2 and Smad 3 as the major downstream regulators. Upon activation, Smad 2 and Smad 3 are phosphorylated and form a complex with Smad 4 that further activates pro-fibrotic gene overexpression [5].

Renal tubular epithelial cells (TEs) have high metabolism and oxygen requirements to maintain normal physiological functions. However, this also makes them prone to hypoxia-induced damage. Renal fibrosis progression is accompanied by anemia and the resulting tissue hypoxia. Hypoxia is an insufficient supply of oxygen and is considered to be a common pathological mechanism ultimately leading to ESRD, as well as tubulointerstitial injury [6,7,8]. Under hypoxic conditions, a crucial transcription factor, hypoxia-inducible factor-1α (HIF-1α), is responsible for regulating cells to hypoxia adaptation [9]. Prolyl-4-hydroxylase 1–3 (PHD1–3) are located in the nucleus and cytoplasm. In normoxia, PHD1–3 utilize molecular oxygen and 2-oxoglutarate as co-substrates to mediate the hydroxylation of HIF-1α at two proline residues, Pro402 and Pro564. The hydroxylation modification enables the von Hippel-Lindau protein (pVHL) to recognize HIF-1α, thereby recruiting an E3 ubiquitin ligase to promote the ubiquitination of HIF-α and its subsequent degradation by the proteasome, ultimately maintaining intracellular HIF-α at low levels [10,11]. Prolyl 4-hydroxylase transmembrane (PHD4) is considered to be the fourth PHD [12]. PHD4 is anchored in the endoplasmic reticulum membrane. PHD4 shares the substrate-interacting residues and the lid structure with other PHDs, while having a unique EF domain. PHD4 hydroxylates the two critical prolines in HIF-1a in vitro with a preference for the C-terminal proline [13,14]. The hydroxylation is blocked, and the expression of HIF-1α is highly elevated in hypoxia. HIF-1α forms a complex with HIF-β and translocates into the nuclei to activate the target genes [15]. HIF-1α promotes renal fibrosis through regulating metabolic reprogramming, activating inflammatory responses, and facilitating EMT. Moreover, the interaction between HIF-1α and fibrosis-related signaling pathways also accelerates the progression of renal fibrosis [16,17,18,19]. HIF-1α forms a transcriptional complex with Smad 3 to promote COL1A2 collagen expression [20,21]. TGF-β enhances the stability of HIF-1α by decreasing the mRNA and protein expressions of prolyl hydroxylase 2 (PHD2) and, therefore, promoting the accumulation of HIF-1α [22]. The knockout of HIF-1α inhibits the progression of tubulointerstitial fibrosis and glomerulosclerosis [6,20]. Therefore, inhibiting HIF-1α and TGF-β/Smad signaling pathways may block the further development of renal fibrosis.

In traditional Chinese medicine (TCM), qi is the driving force for the generation and circulation of blood. Blood is the basis and carrier of qi. When qi is insufficient, the generation and circulation of blood are hindered, resulting in a microenvironment of ischemia and hypoxia in the local area. This affects cellular metabolism and promotes the occurrence of inflammation. Long-term chronic inflammation eventually results in damage to kidney functions. Thus, tonifying qi and promoting blood circulation may help to alleviate hypoxia and the progression of CKD. Dangguibuxue decoction (DD) is a well-known TCM for tonifying qi and nourishing blood in China. DD contains *Angelica sinensis* (Oliv.) Diels (AS) and *Astragalus membranaceus* (Fisch.) Bge. (AM) at a ratio of 1:5. It has shown therapeutic effects in diabetic nephropathy, renal anemia, and non-dialysis diabetes nephropathy complicated by renal anemia by improving renal functions and reducing abnormal ECM accumulation [23,24,25]. AM exerts anti-inflammatory and immune-enhancing effects and is regarded as a good qi tonic medicine in clinical practice. Studies have shown that AM exhibited renal protective effects by inhibiting tubular EMT and renal fibrosis through the TGF-β/Smad pathway [26,27]. AS has been shown to nourish blood and promote blood circulation [28,29]. AS polysaccharides could stimulate renal erythropoietin (EPO) production by increasing the expression of HIF-2α, while reducing the expression of nuclear factor kappa-B (NF-κB), recombinant GATA Binding Protein 2 (GATA2), and pro-inflammatory cytokines to relieve adenine-induced anemia and renal damage in CKD rats [30]. In addition, AS has shown dose- and time-dependent reno-protective effects in patients with CKD, according to a longitudinal cohort study [31].

Studies have attempted to clarify the mechanisms and identify the active components of DD in alleviating renal fibrosis [32,33]. However, relevant studies on the mechanisms of DD in the treatment of renal fibrosis have not yet offered an in-depth exploration from the perspective of traditional clinical application. Here, we systematically compared the anti-fibrotic effects of AS, AM, and DD through renal functions, histopathology, and fibrotic marker expressions. In addition, the active ingredients of DD were detected using UPLC-MS/MS and their anti-fibrotic activities were further screened by molecular docking and verified by in vitro assays. This study aims to explore the mechanisms by which DD inhibits fibrosis based on its traditional medicinal functions.

## 2. Results

### 2.1. Detection of Chemical Components Using UPLC-MS/MS

**Figure 1:**
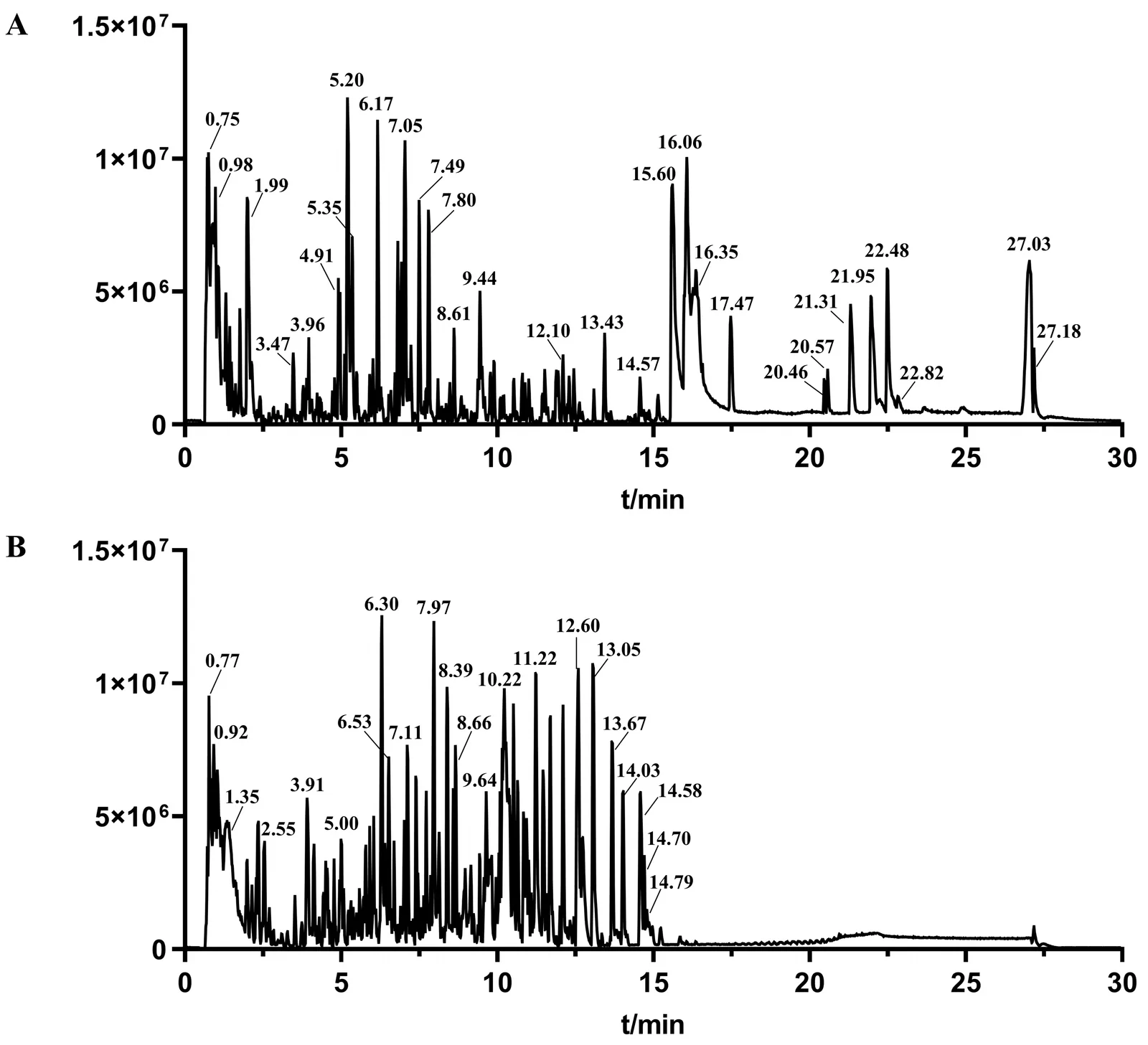
Chromatograms of Dangguibuxue decoction (DD). (**A**) Positive ion mode. (**B**) Negative ion mode.

To explore the active components in DD, the chemical components were first determined by UPLC-MS/MS (Figure 1). In total, 79 compounds were identified, including 21 flavonoids, 8 saponins, 14 phthalides, 14 organic acids, 6 phenylpropanoids, 1 alkaloid, and 15 other compounds. Among the 21 flavonoids, there were 5 flavonols, 12 isoflavones, 2 isoflavans, 1 pterocarpan, and 1 chalcone compound (Table S1). Kaempferol, calycosin, isoquiritigenin, isomucronulatol, rhamnocitrin, rutin, ononin, formononetin, quercetin, and their derivatives were detected and identified. Calycosin was eluted at 5.21 min and identified with the parent ion of *m*/*z* 285.0757 [M + H]^+^ and fragment ions of *m*/*z* 270.0519 [M + H − CH_3_]^+^ and *m*/*z* 253.0494 [M + H − CH_3_ − OH]^+^, respectively. Saponin compounds were mainly derived from *Astragalus membranaceus*, and representative compounds include astragaloside I, II, III, IV, and acetylastragaloside I. Astragaloside IV was observed at *m*/*z* 807.4492 [M + Na]^+^. After collision, fragment ions were obtained at *m*/*z* 587.3938 [M + H − Glu − H_2_O]^+^, *m*/*z* 569.3822 [M + H − Glu − 2H_2_O]^+^, *m*/*z* 473.3610 [M + H − Glc − C_5_H_8_O_4_]^+^, *m*/*z* 455.3507 [M + H − Glc − C_5_H_10_O_5_]^+^, and *m*/*z* 437.3407 [M + H − Glc − C_5_H_10_O_5_ − H_2_O]^+^. Phthalide compounds and their derivatives, mainly existing in *Angelica sinensis,* were also detected. Z-ligustilide and its isomers showed similar cleavage pattern in mass spectrum, and were characterized with the same parent ion *m*/*z* 191.1065 [M + H]^+^ and fragment ions of *m*/*z* 163.1104 [M + H − CO]^+^, *m*/*z* 149.0581 [M + H − CH_2_CO]^+^, *m*/*z* 145.1002 [M + H − CO − H_2_O]^+^, and *m*/*z* 135.1167 [M + H − CH_2_CO − CH_2_]^+^, respectively. In addition, other compounds, such as organic acids, amino acids, monosaccharides, and nucleosides, were also detected in DD.

### 2.2. Network Pharmacology Analysis Predicts Potential Targets

**Figure 2:**
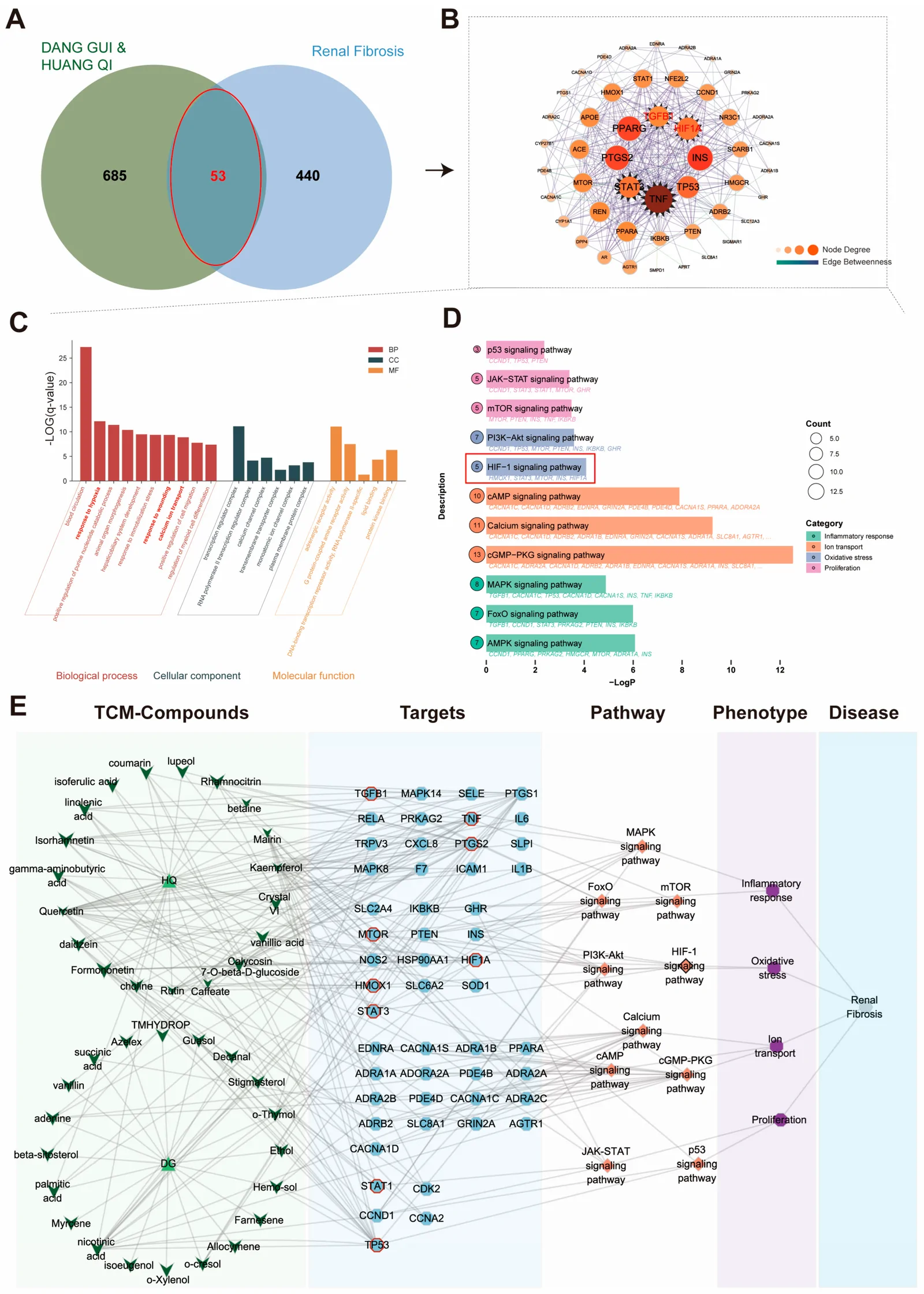
Network pharmacology analysis of DD in the treatment of renal fibrosis (RF). (**A**) Venn diagram of DD and RF targets. (**B**) PPI network of core targets of DD and RF. (**C**) GO functional enrichment items. (**D**) Related KEGG pathways. (**E**) The “components–targets–disease” network of DD and RF. The green arrows represent the active components in DD. The blue circles indicate the potential targets. The orange diamonds denote the potential signaling pathways. The purple circles indicate related phenotypes.

In order to explore the potential mechanism of DD against renal fibrosis (RF), network pharmacology analysis was conducted. A total of 685 targets of 103 components in DD were predicted online. In addition, 440 targets related to renal fibrosis were extracted from databases such as GeneCards, OMIM, TTD, and DisGeNET. The predicted therapeutic targets of each component were collected. The top ten components and targets are listed in Table S2 and Table S3, respectively. As shown in Figure 2A, 53 targets common to DD and RF were identified.

Constituents including quercetin, kaempferol, 7-O-methylisomucronulatol, formononetin, and rutin had strong associations with RF. PPI analysis indicated that TNF, HIF-1α, TGF-β, PPARG, TP53, PTGS2, INS, and STAT were the potential core targets of DD in treating RF (Figure 2B). GO functional analysis suggested that DD may exert antioxidant and anti-inflammatory effects to attenuate renal fibrosis by modulating TNF, STAT1, NFKBIA, STAT3, and TGF-β. In addition, multiple biological processes, such as blood circulation, response to hypoxia, and response to wounding, were also involved (Figure 2C). KEGG enrichment analysis suggested that the PI3K-Akt signaling pathway, HIF-1α signaling pathway, MAPK signaling pathway, mTOR signaling pathway, AMPK signaling pathway, calcium signaling pathway, and cGMP-PKG signaling pathway were involved in the treatment of DD for renal fibrosis (Figure 2D). A “components–targets–renal fibrosis” interaction network was constructed to analyze the potential therapeutic targets of DD in renal fibrosis based on the 53 overlapping targets (Figure 2E). Considering the role of hypoxia in the progression of renal fibrosis, HIF-1α and TGF-β/Smad signaling pathways were chosen for further analysis.

### 2.3. DD Improves Renal Functions

**Figure 3:**
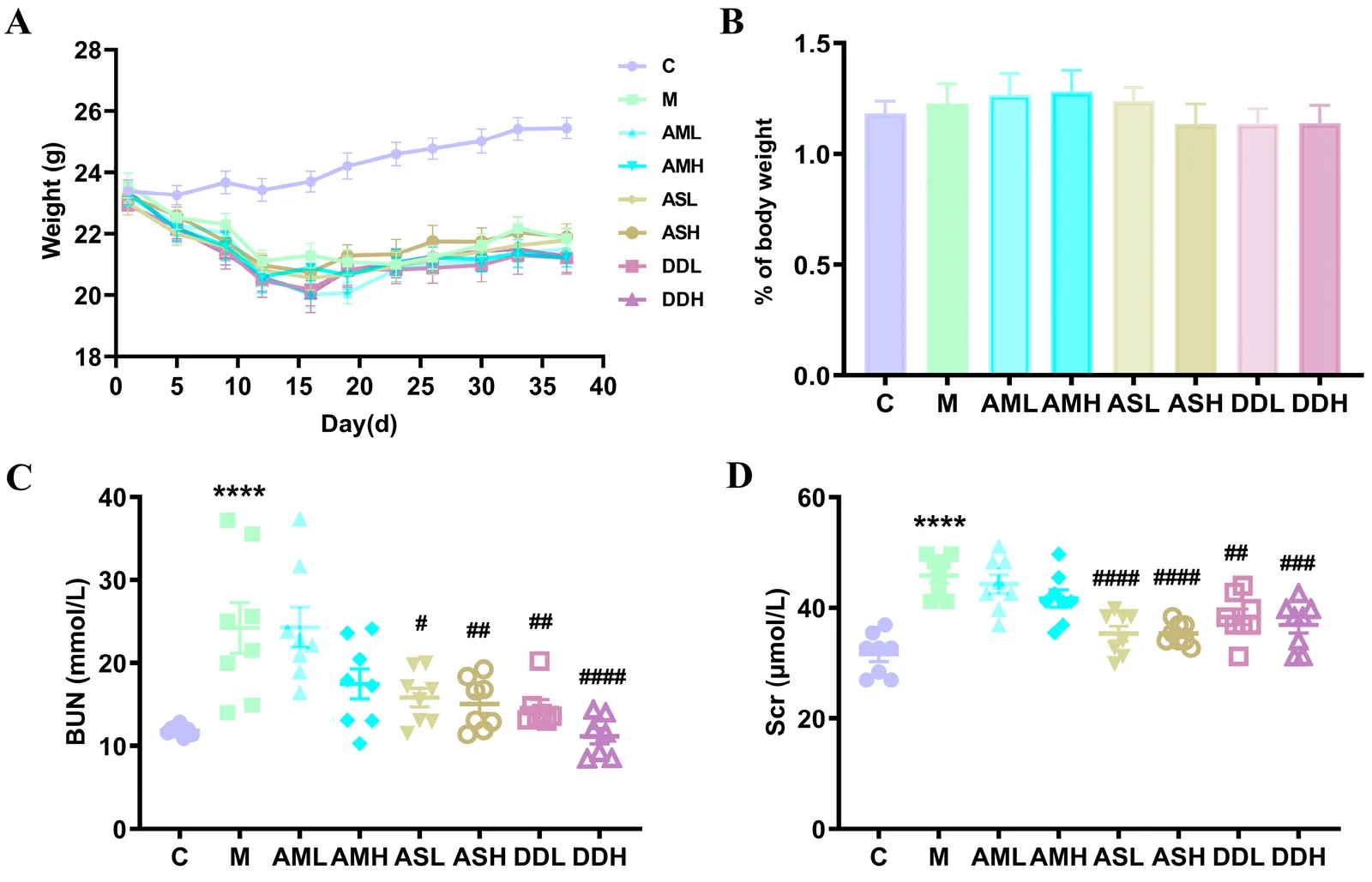
The body weights and renal functions in drug treated mice. (**A**) The body weight changes in mice treated with test drugs, including AA I, AML, AMH, ASL, ASH, DDL, and DDH, respectively. (**B**) The relative weights of renal tissue in mice after administration of test drugs. (**C**) Level changes in serum BUN of mice treated with test drugs. (**D**) Level changes in serum Cre of mice treated with test drugs. Statistical comparisons: ****, *p* < 0.0001 vs. control; ^#^, *p* < 0.05; ^##^, *p* < 0.01; ^###^, *p* < 0.001; ^####^, *p* < 0.0001 vs. model (*n* = 8).

The body weights of mice in the control group increased steadily during the experimental period. However, the body weights of mice administered with AA I decreased significantly. Although weight increased slightly after discontinuing the treatment with AA I, mice in the AA I and treatment groups experienced significant weight loss. No obvious difference was observed in body weights between AA I and medication groups (Figure 3A). Renal tissues were weighed to assess the effect of AM, AS, and DD on the kidney coefficient under AA I toxic conditions. ASH, DDL, and DDH decreased the elevation of the renal weight index to some extent; however, there was no significant change between each group (Figure 3B).

A significant increase in serum BUN in the AA I group was observed (*p* < 0.0001). After treatment with ASL, ASH, DDL, and DDH, the serum BUN was dramatically decreased (*p* < 0.05, *p* < 0.01, or *p* < 0.0001) (Figure 3C). In addition, the decrease showed a certain concentration dependence for AS and DD. There was a significant increase in serum Cre after AA I administration (*p* < 0.0001). However, the serum Cre in ASL, ASH, DDL, and DDH groups was remarkably decreased (*p* < 0.01, *p* < 0.001, or *p* < 0.0001) (Figure 3D). These results indicate that AS and DD effectively improved renal functions of AA I-induced renal fibrosis mice.

### 2.4. DD Alleviates AA I-induced Renal Fibrosis and Reduces Abnormal ECM Accumulation

**Figure 4:**
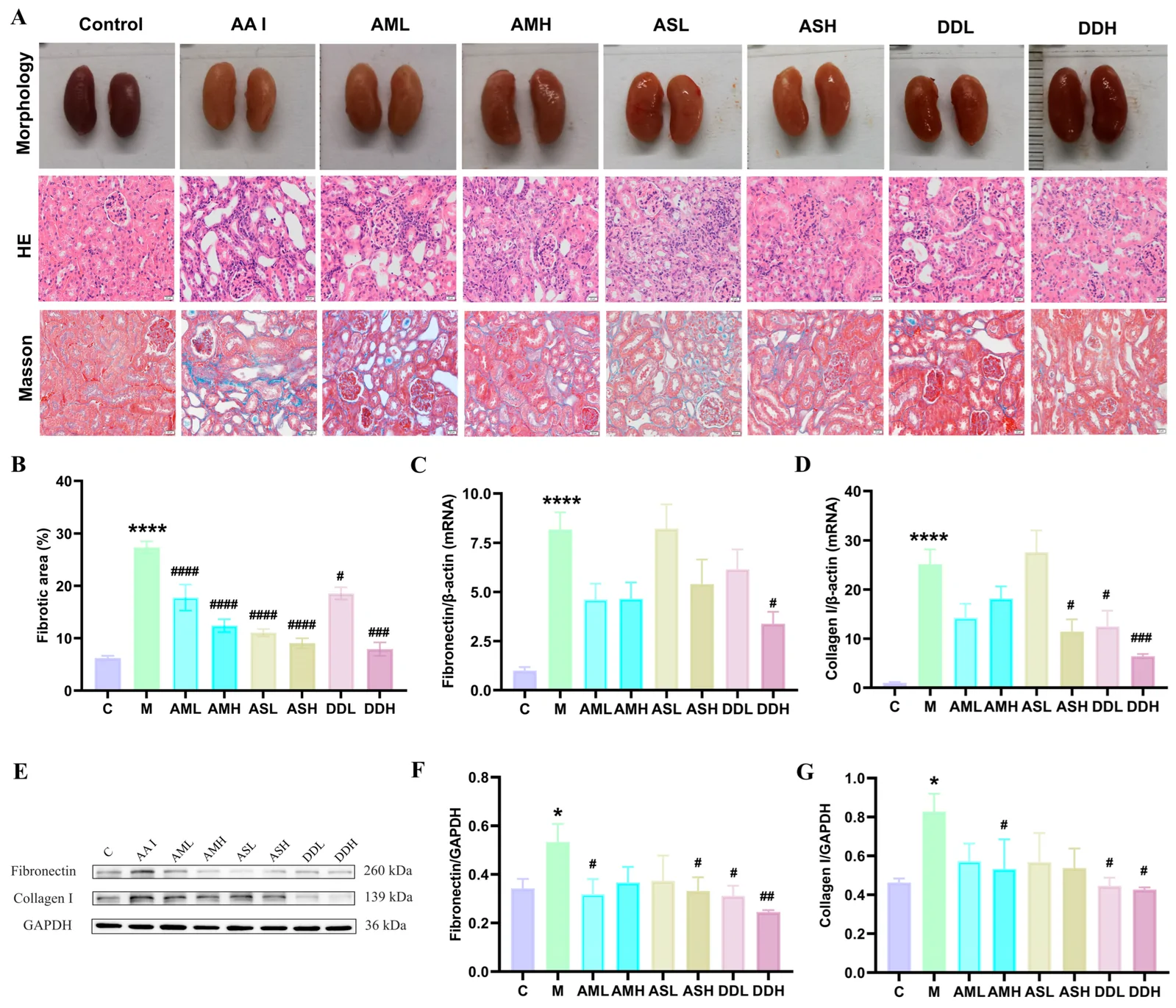
Pathological examination of renal tissues. (**A**) Renal tissue morphology, H&E staining and Masson staining of mice in control, AA I, AML, AMH, ASL, ASH, DDL, and DDH groups, respectively. Images were taken at a magnification of 400×. Scale bar, 20 μm. (**B**) The ratio of renal fibrotic areas in control, AA I, AML, AMH, ASL, ASH, DDL, and DDH groups, respectively. Masson staining images were analyzed with Image J software (1.54). (**C**,**D**) The mRNA expression of fibronectin and collagen I. (**E**–**G**) The protein expression of fibronectin and collagen I. Data are presented as the mean ± SD. *, *p* < 0.05; ****, *p* < 0.0001 vs. control; ^#^, *p* < 0.05; ^##^, *p* < 0.01; ^###^, *p* < 0.001; ^####^, *p* < 0.0001 vs. model (*n* = 9 for fibrotic area, *n* = 6 for mRNA expression, and *n* = 3 for Western blotting).

The histopathological changes in renal tissues in different treatment groups were estimated by H&E staining and Masson staining. The renal tissues of control mice showed no obvious abnormalities. However, significant pathological changes were observed in the renal tissues of mice that received AA I administration. Typical pathological changes in renal tissues included extensive necrosis of renal tubules, disordered renal tubular arrangement, degeneration, dilated lumens, and inflammatory cell infiltration. In addition, some proximal tubules showed the detachment of the brush border. AMH, ASH, and DDH improved renal tubule necrosis and arrangement. Masson staining revealed a significant increase in interstitial fibrosis area in renal tissues of the AA I group (*p* < 0.0001), whereas the fibrotic areas in the treatment groups showed varying degrees of reduction (*p* < 0.05, *p* < 0.001, or *p* < 0.0001), among which DDH demonstrated the optimal therapeutic effect (Figure 4A,B).

Significant increases in the mRNA and protein expression of collagen I and fibronectin in the AA I group were observed (*p* < 0.05 or *p* < 0.0001), indicating abnormal ECM accumulation in mice after intraperitoneal injection of AA I. The mRNA expression of collagen I in the ASH, DDL, and DDH groups (*p* < 0.05 or *p* < 0.001) and fibronectin in the DDH group was significantly downregulated (*p* < 0.05). In addition, the protein expressions of collagen I in the AMH, DDL, and DDH groups and fibronectin in the AML, ASH, DDL, and DDH groups were also reduced (*p* < 0.05 or *p* < 0.01). These findings illustrated that DD downregulated AA I -induced ECM accumulation (Figure 4C–G).

### 2.5. DD Affects the HIF-1α and TGF-β/Smad Signaling Pathways

**Figure 5:**
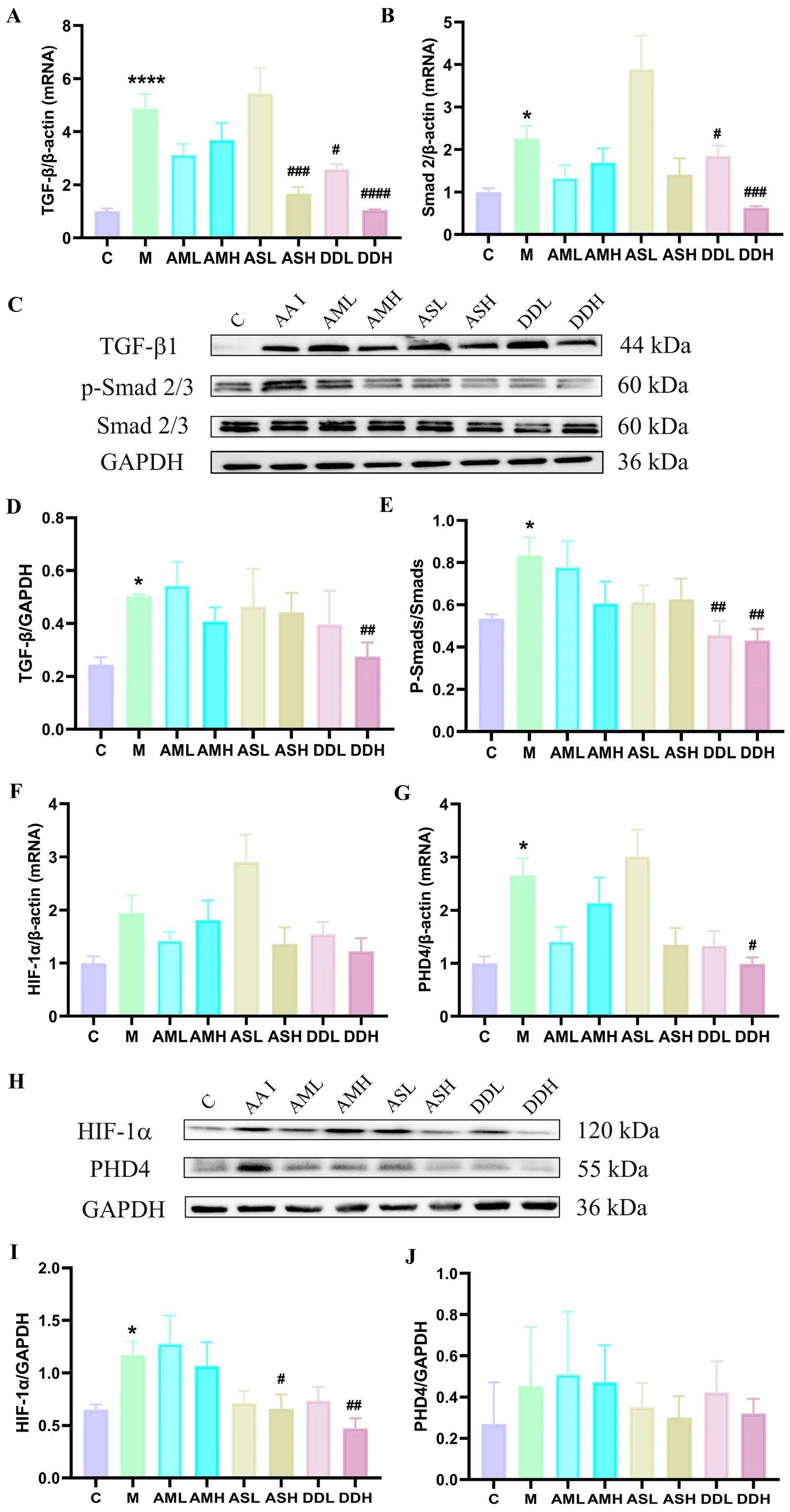
DD attenuated AA I-induced renal fibrosis. (**A**,**B**) The mRNA expression of TGF-β and Smad 2. (**C**–**E**) The protein expression of TGF-β, Smad 2/3, and p-Smads2/3. (**F**,**G**) The mRNA expression of HIF-1α and PHD4. (**H**–**J**) The protein expression of HIF-1α and PHD4. *, *p* < 0.05; ****, *p* < 0.0001 vs. control. ^#^, *p* < 0.05; ^##^, *p* < 0.01; ^###^, *p* < 0.001; ^####^, *p* < 0.001 vs. model (*n* = 6 for mRNA expression and *n* = 3 for Western blotting).

Compared with the control group, the mRNA and protein expression of TGF-β and Smad2 in the AA I group were significantly increased (*p* < 0.05 or *p* < 0.0001), consistent with histopathological changes in AA I-induced renal fibrosis (Figure 5A–E). There was a remarkable decrease in the mRNA expression of TGF-β in ASH, DDL, and DDH groups (*p* < 0.05, *p* < 0.001, or *p* < 0.0001). Additionally, a significant decrease in the protein expression of TGF-β was observed in the DDH group (*p* < 0.01). Evident decreases in the mRNA expression of Smad 2 and protein expression ratios of p-Smad 2/3 to Smad2/3 were observed in the DDL and DDH groups (*p* < 0.05, *p* < 0.01, or *p* < 0.001).

No significant change in the mRNA expression of HIF-1α was observed between each group (Figure 5F). An obvious increase in mRNA expression of PHD4 in the AA I group was observed (*p* < 0.05); however, this increase was reversed by DDH (*p* < 0.05) (Figure 5G). HIF-1α protein expression was remarkably downregulated by ASH and DDH (*p* < 0.05, or *p* < 0.01) (Figure 5H,I). No obvious difference in the protein expression of PHD4 was observed (Figure 5J).

### 2.6. Screening of Active Components by Molecular Docking

**Figure 6:**
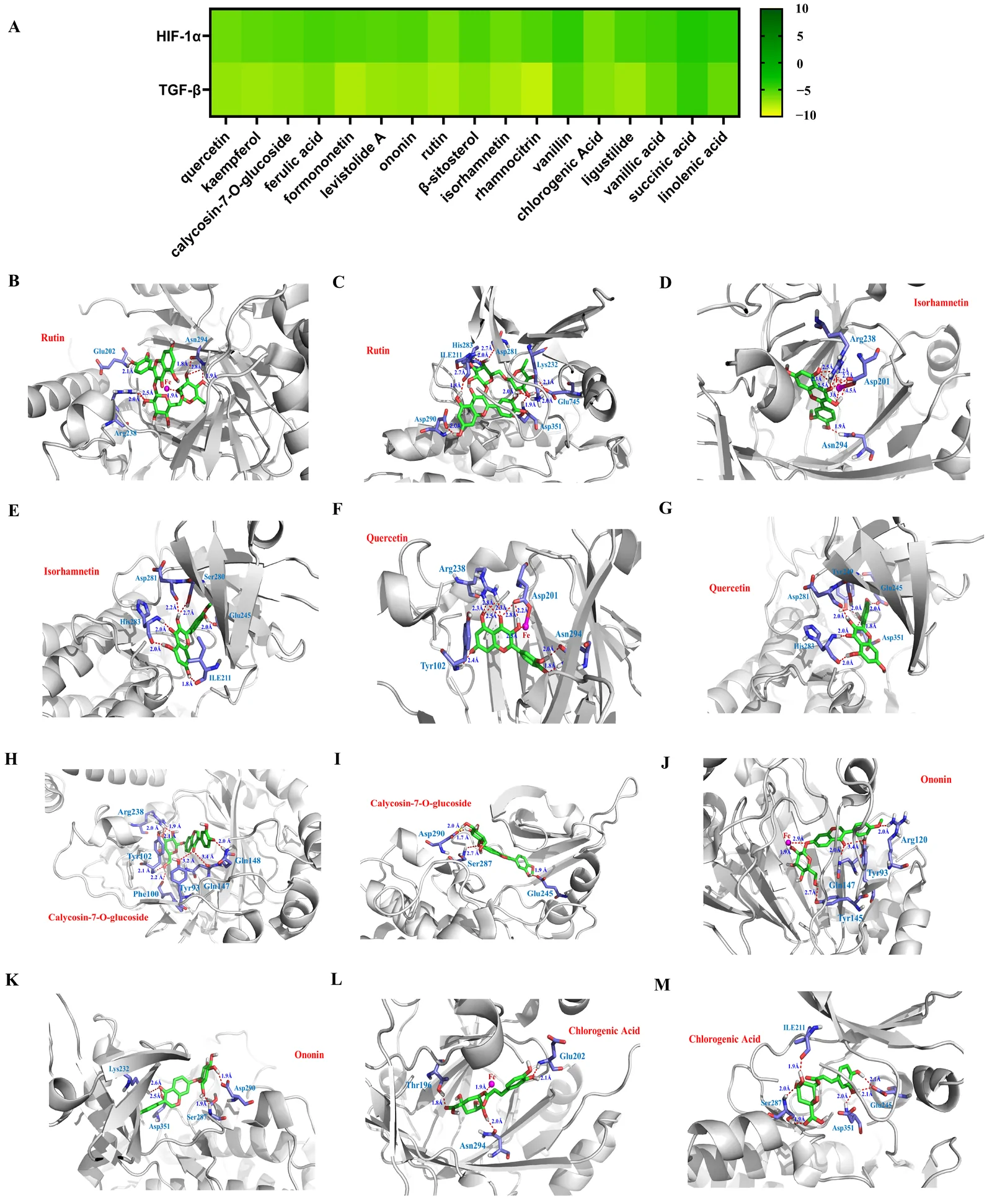
Heat map and molecular docking results. (**A**) Heat map of molecular docking results of 19 compounds. (**B**–**M**) Molecular docking images of rutin, isorhamnetin, quercetin, calycosin-7-O-glucoside, ononin, and chlorogenic acid, respectively.

Through network pharmacology analysis, 17 compounds were found to potentially target HIF-1α or TGF-β. To further screen the bioactive components of DD, molecular docking was applied, and the binding energies of test compounds were obtained. Results showed that 12 compounds had high binding affinities with binding energies < −5.0 kcal/mol (Figure 6A). Among these, rutin, isorhamnetin, calycosin-7-O-glucoside, chlorogenic acid, quercetin, and ononin were implied to have better anti-fibrotic effects, owing to their lower binding energies (Figure 6B–M). In particular, the binding energies of rutin reached −6.30 kcal/mol and −7.57 kcal/mol, respectively. Rutin formed six hydrogen bonds with Glu202, Arg238, and Asn294 of the factor inhibiting HIF-1α, with bond lengths between 1.8 and 2.8 Å. In addition, it formed nine hydrogen bonds with ILE211, Lys232, Asp281, His283, Asp290, Asp351, and Glu745 of TGF-βRI, with bond lengths between 1.8 and 2.7 Å.

### 2.7. In Vitro Inhibition of Renal Fibrosis by Active Components

**Figure 7:**
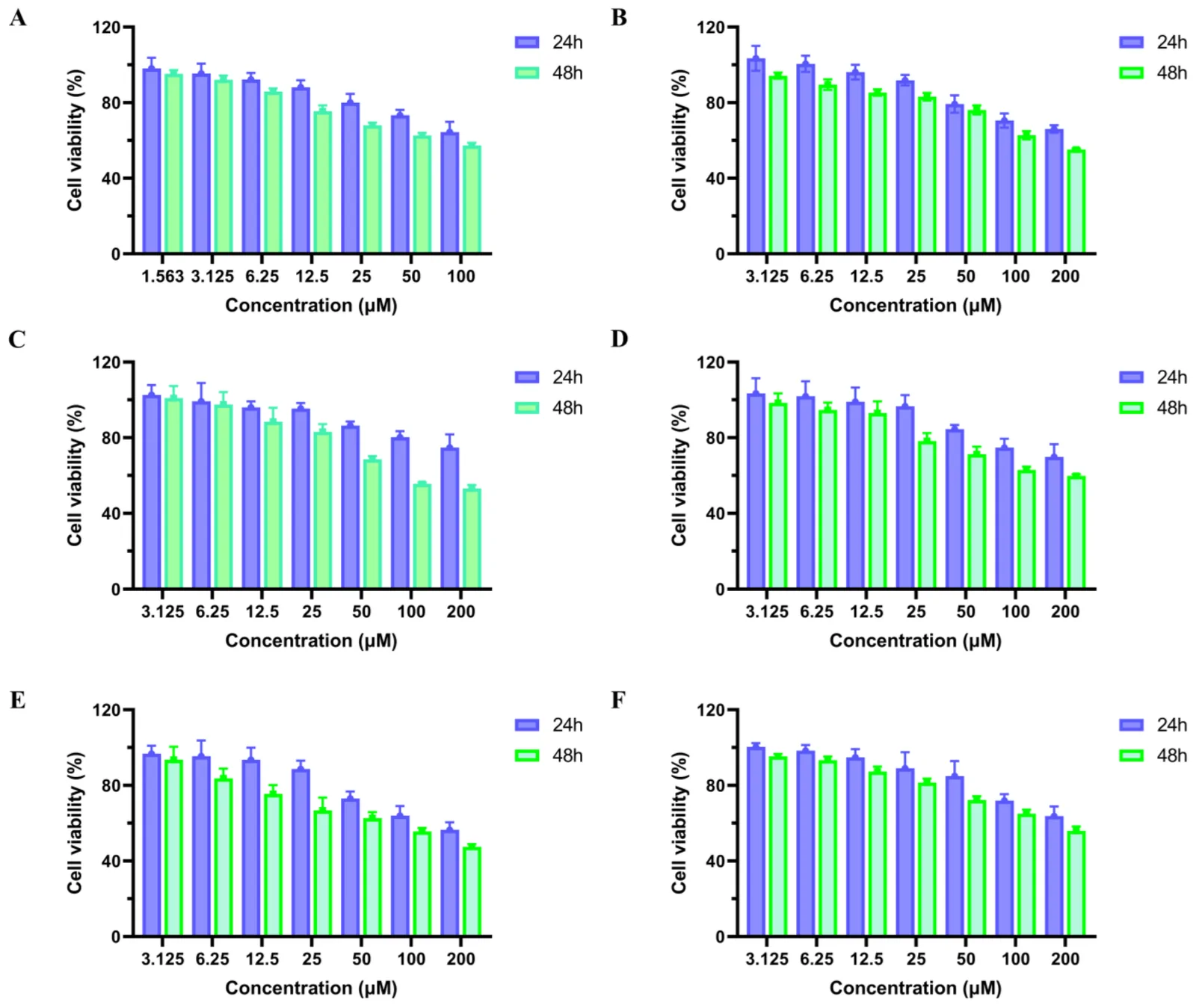
Cell viability assay (CCK-8) results of (**A**) rutin, (**B**) isorhamnetin, (**C**) calycosin-7-O-glucoside, (**D**) chlorogenic acid, (**E**) quercetin, and (**F**) ononin after incubation with HK-2 cells at different concentrations for 24 and 48 h, respectively. Data were obtained from 6 replicates.

To further screen the active constituents in DD, the top six compounds in terms of low binding energies—that is, rutin, isorhamnetin, calycosin-7-O-glucoside, chlorogenic acid, quercetin, and ononin—were chosen for the in vitro validation of anti-fibrotic effects. Drug toxicity tests on HK-2 cells were first conducted through a CCK8 assay. The results showed that all compounds exhibited low cell toxicity on HK-2 cells, with IC_50_ values above 200 μM after incubation for 24 or 48 h (Figure 7A–F).

**Figure 8:**
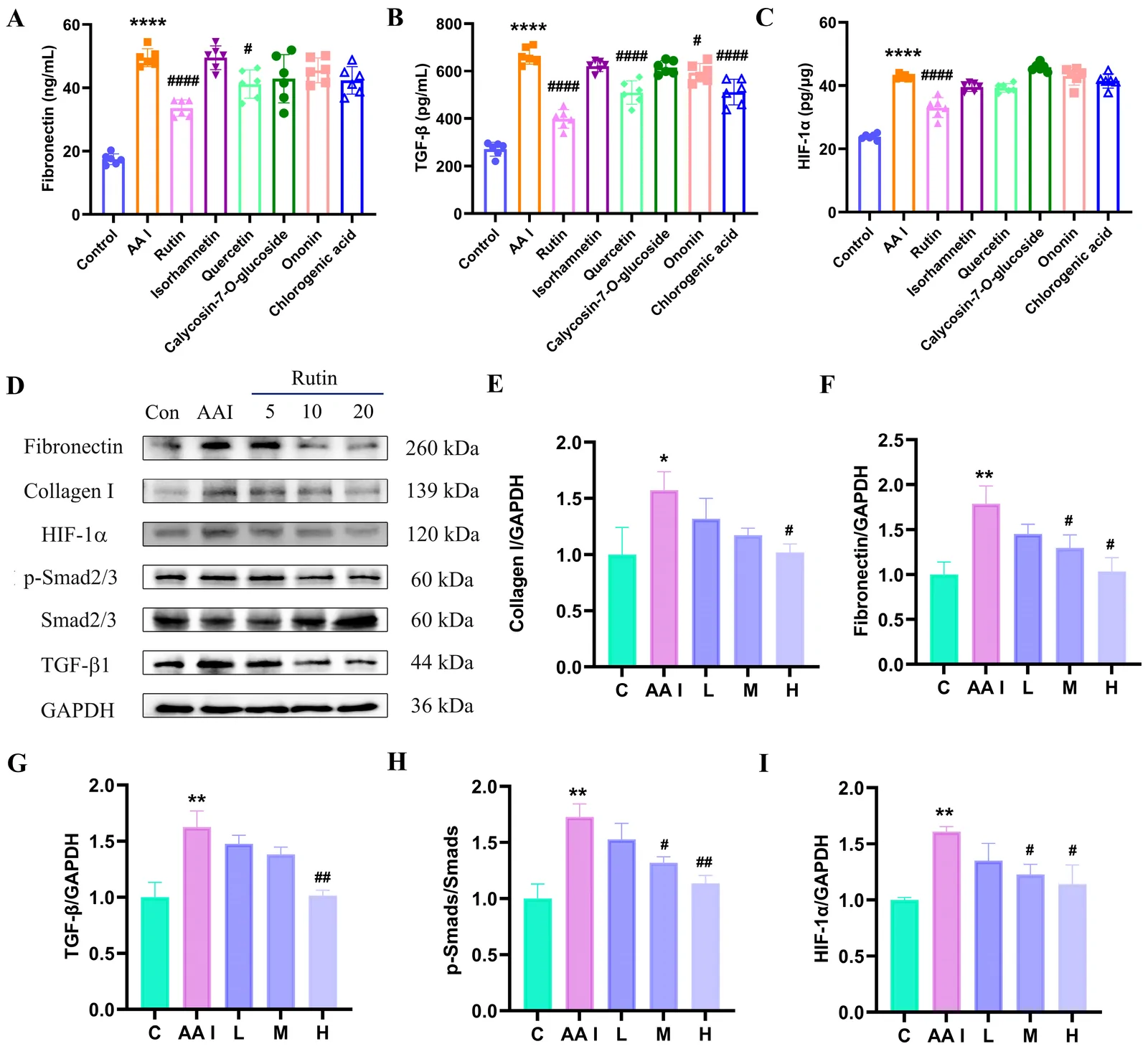
Anti-fibrotic effect evaluation of 6 active compounds through in vitro assays. (**A**–**C**) The expressions of HIF-1α, TGF-β, and fibronectin in cell lysate or supernatant after treatment with AA I (10 μM) and/or 6 compounds. (**D**–**I**) Expressions of HIF-1α, TGF-β, fibronectin, collagen I, Smad 2/3, p-Smad 2/3, and GAPDH in HK-2 cells after treatment with rutin at different concentrations. C: Control group; L, M, and H: Rutin at 5, 10, and 20 μM, respectively. *, *p* < 0.05; **, *p* < 0.01; ****, *p* < 0.0001 vs. control. ^#^, *p* < 0.05; ^##^, *p* < 0.01; ^####^ *p* < 0.0001 vs. model (*n* = 6 for ELISA and *n* = 3 for Western blotting).

Cellular fibrosis was first induced by incubation with AA I (10 μM) for 48 h. Enzyme-linked immunosorbent assay (ELISA) results showed remarkably increased expressions of HIF-1α, TGF-β, and fibronectin in the AA I group (*p* < 0.0001), which indicated the progress of fibrosis (Figure 8A–C). Rutin, quercetin, chlorogenic acid, and ononin significantly decreased the protein expression of TGF-β in HK-2 cells (*p* < 0.05 or *p* < 0.0001). Fibronectin expression was reduced after treatment with rutin and quercetin (*p* < 0.0001 or *p* < 0.05). Additionally, rutin significantly reduced the expression of HIF-1α in HK-2 cells (*p* < 0.0001). However, there were no significant changes in the expression of HIF-1α, TGF-β, and fibronectin after treatment with isorhamnetin or calycosin-7-O-glucoside in HK-2 cells.

Among these compounds, rutin showed the best anti-fibrotic effect in HK-2 cells. Therefore, the concentration-dependent inhibition of rutin on the expression levels of collagen I, fibronectin, TGF-β, Smad 2/3, and HIF-1α was further assessed using Western blotting assays. As shown in Figure 8D–I, the expressions of the above proteins in HK-2 cells underwent significant increases after AA I treatment (*p* < 0.05 or *p* < 0.01), whereas the expressions of collagen I, fibronectin, TGF-β, p-Smad/Smad, and HIF-1α showed a concentration-dependent decline after incubation with rutin, with significant differences at 10 μM and/or 20 μM of rutin (*p* < 0.05, or *p* < 0.01). These findings demonstrated that rutin may be a potential bioactive compound of DD in the attenuation of renal fibrosis by potentially targeting the HIF-1α and TGF-β/Smad pathways in HK-2 cells.

**Figure 9:**
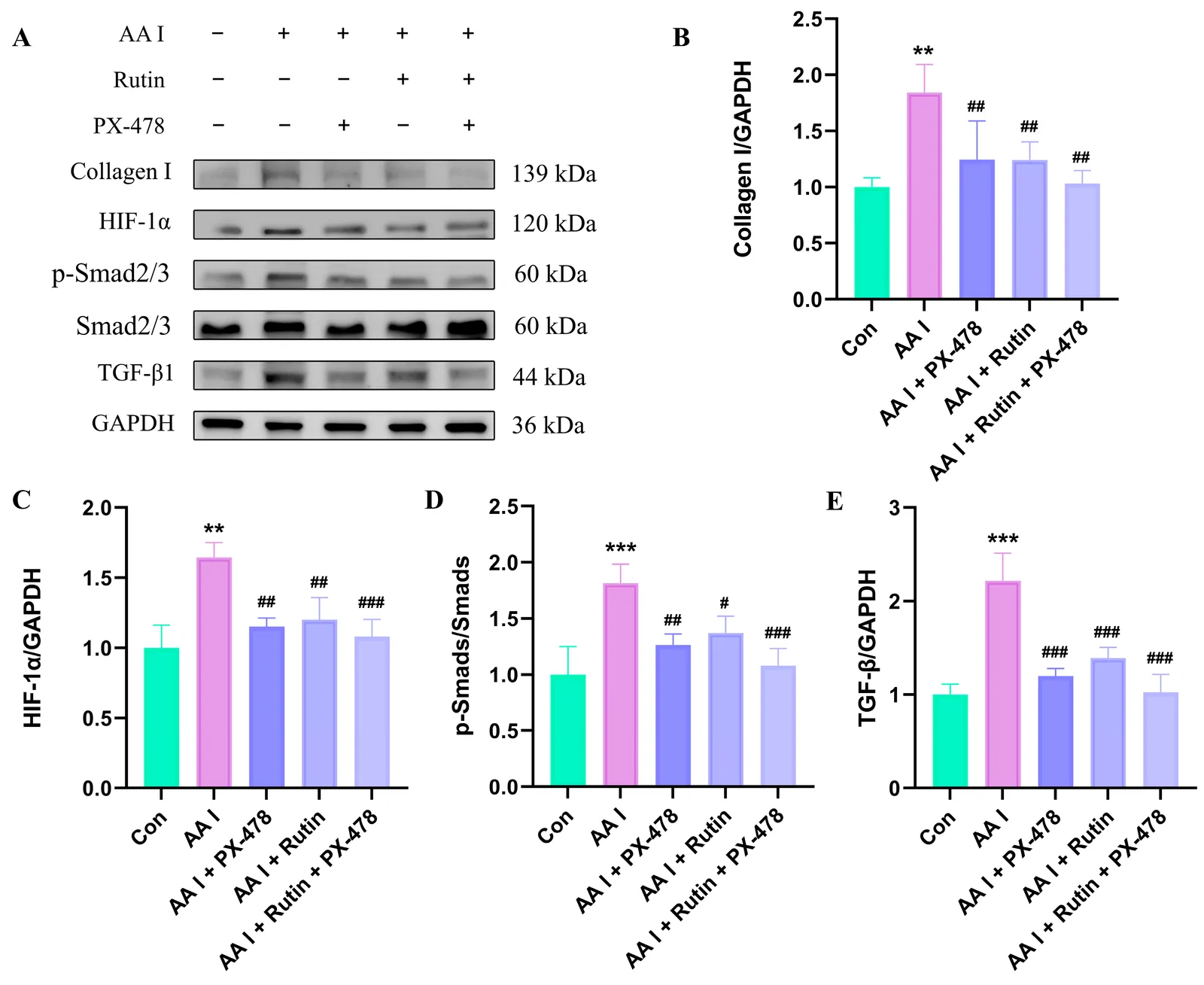
Inhibition of the protein expression of HIF-1α in HK-2 cells. (**A**–**E**) Expressions of HIF-1α, TGF-β, collagen I, Smad 2/3, p-Smad 2/3, and GAPDH in HK-2 cells after treatment with AA I, rutin, PX-478 or rutin with PX-478. **, *p* < 0.01; ***, *p* < 0.001 vs. control. ^#^, *p* < 0.05; ^##^, *p* < 0.01; ^###^ *p* < 0.001 vs. model (*n* = 3 for Western blotting).

Next, we applied an inhibitor of HIF-1α, PX-478, to validate the inhibitory effect of rutin in HK-2 cells. Results showed that the protein expressions of collagen I, HIF-1α, TGF-β, and p-Smads/Smads were significantly increased in the AA I group (*p* < 0.01, *p* < 0.001). However, after treatment with PX-478 or rutin, the increasing trends of collagen I, HIF-1α, TGF-β, and p-Smads/Smads were largely reversed (*p* < 0.05, *p* < 0.01, *p* < 0.001). There was no significance in the protein expression of collagen I, HIF-1α, p-Smads/Smads, and TGF-β between the AA I + PX-478 and AA I + Rutin group, supporting the possible involvement of HIF-1α. Additionally, no significant differences in protein expression of collagen I, HIF-1α, and p-Smads/Smads were observed between the AA I + Rutin + PX-478 and AA I + Rutin group, except for a further decrease in TGF-β (*p* < 0.05). The combination with PX-478 did not result in a further significant decrease in HIF-1α, indicating that rutin and PX-478 may act through a similar HIF-1α signaling pathway in HK-2 cells (Figure 9A–E).

## 3. Discussion

CKD has attracted worldwide attention as a major threat to human health. Renal fibrosis is the core pathological hallmark underlying the progression of CKD to ESRD. Renal fibrosis is dynamically driven by complicated factors and multiple pathways. Syndrome differentiation treatment and multiple targets have made TCM a hot topic in research on CKD treatment. DD, which comprises *Angelica sinensis* (Oliv.) Diels (AS) and *Astragalus membranaceus* (Fisch.) Bge. (AM), was first recorded in Differentiation on Endogenous and Exogenous Diseases in the Jin Dynasty (1115–1234). AM is usually used to invigorate qi, and AS is often used to nourish the blood and promote blood circulation. DD has shown positive effects in the clinical treatment of CKD. Nevertheless, the potential mechanisms and active constituents are still unclear.

Network pharmacology has become a valuable tool in predicting potential active components, targets, and the underlying mechanisms for TCM. Herein, a total of 685 targets of 103 components in DD and 440 targets related to renal fibrosis were extracted from databases network pharmacology analysis. Through PPI, GO, and KEGG enrichment analysis, the HIF-1α, PI3K-Akt, MAPK, calcium, and TGF-β signaling pathways were indicated to potentially be involved in the anti-fibrotic effect of DD. The PI3K-Akt signaling pathway and the MAPK signaling pathway, as upstream pathways, can upregulate the transcription and protein expression of HIF-1α [34,35]. Therefore, these pathways suggest a possible association with hypoxia. HIF-1α is a vital transcription factor in hypoxia. It is well known that HGB serves as the primary oxygen-transporting carrier in the blood, facilitating the reversible binding and delivery of oxygen to tissues. Clinical data show that CKD is often accompanied by anemia [36]. Anemia leads to a decrease in the oxygen-carrying ability of blood, causing insufficient oxygen supply in the kidneys [37]. In this research, DD increased RBC, HGB, HCT, MCV, and RDW in the blood and the protein expression of EPO, which is in agreement with its traditional blood-nourishing effect. But whether the improvement of anemia leads to the alleviation of hypoxia requires further experiments to prove.

Recently, more and more evidence is supporting the contributive role of hypoxia in renal fibrosis [38,39,40]. Hypoxia triggers fibroblast activation and ECM deposition [41]. In addition, HIF-1α interacts with diverse profibrotic signaling pathways to further activate renal fibrosis [42]. Specifically, HIF-1α can form a complex with Smad3 to promote TGF-β-induced COL1A2 collagen expression and interstitial fibrosis [43]. In turn, TGF-β increases the expression of HIF-1α (dependent on the kinase activity of TβRI) and stabilizes HIF-1α by decreasing the mRNA and protein levels of PHD2 [21]. The mutual feedback regulation between HIF-1α and TGF-β promotes the progression of renal fibrosis synergistically [43,44]. Therefore, inhibiting HIF-1α and TGF-β is an important strategy in renal fibrosis treatment [4,45]. In this research, the protein expressions of ECM biomarkers were significantly increased in both AA I-treated HK-2 cells and mice. However, this increasing trend was suppressed by AM, AS, and DD. Comparatively, DD exerted better attenuating efficacy than AS or AM. DD improved renal functions by reducing serum BUN and Cre levels. Histologically, DD effectively decreased the areas of cell necrosis and fibrosis, and significantly decreased the mRNA and protein expression of collagen I and fibronectin. PHDs play an important role in regulating HIF-1α abundance and maintaining the oxygen balance through the hydroxylation of HIF-1α and by promoting polyubiquitination and proteasomal degradation. Among these, PHD2 is the critical oxygen sensor setting low steady-state levels of HIF-1α in normoxia [46]. However, no statistically significant change in PHD2 expression was observed in this study. PHD4, as an isoform of PHDs, was observed to be greatly increased in HEK293 cells in hypoxia [14]. Destruction of renal glomeruli and proteinuria resulting from severe impairment of renal function were observed in PHD4-deficient zebrafish and PHD4 (PHD4, also known as P4HTM, *P4h-tm^−^^/^^−^*) knockout mice, which indicated that PHD4 may play a role in maintaining the normal structure and function of the kidneys [47,48]. Additionally, PHD4 is also related to *Helicobacter pylori-induced* chronic gastritis, atherosclerotic plaques, and syndromic obesity [49,50,51]. Herein, the mRNA expression of PHD4 was significantly increased in AA I-induced renal fibrosis mice and reduced by DDH. Whether this altered expression is hypoxia-induced or acts independently remains to be further investigated. In addition, DD reduced the mRNA expression of TGF-β and Smad 2 and the protein expression of HIF-1α, TGF-β, and the ratio of p-Smads/Smads. These findings suggest that the attenuation of DD in AA I-induced renal fibrosis may be associated with the inhibition of HIF-1α and TGF-β/Smad signaling pathways.

Herbal medicines contain numerous bioactive components. The verification of active ingredients is beneficial for improving therapeutic efficacy and ensuring the quality control of drugs. Furthermore, it also facilitates the research and development of new drugs. A preliminary screening of the anti-fibrotic ability of 17 compounds obtained from network pharmacology analysis was conducted by molecular docking in this research work. Among these compounds, rutin, isorhamnetin, calycosin-7-O-glucoside, chlorogenic acid, quercetin, and ononin showed strong binding affinity to HIF-1α and TGF-β. Quercetin, isorhamnetin, chlorogenic acid and rutin have shown good therapeutic effects against nephropathy in previous studies [52,53,54,55]. In this research, rutin exhibited an anti-fibrotic effect by reducing the abnormal accumulation of collagen I and fibronectin and decreasing the protein expressions of TGF-β and p-Smads/Smads in HK-2 cells, in accordance with the findings of previous studies [56,57]. Further experiments indicated that rutin showed a similar inhibitory effect to PX-478, a specific HIF-1α inhibitor, which provided further evidence on the possible involvement of HIF-1α. In addition, the combination of PX-478 did not significantly decrease the expression of HIF-1α compared with the rutin group, indicating that rutin may act on a similar HIF-1α signaling pathway.

There are several limitations to this study. First, the therapeutic effect of DD was evaluated in AA I-induced renal fibrosis, but whether it is effective for other fibrotic diseases will depend on further studies. In addition, only HK-2 proximal tubular epithelial cells were used for the in vitro study. Further experiments on renal interstitial fibroblasts or myofibroblasts were needed to fully characterize its regulatory function on interstitial fibroblast activation and extracellular matrix accumulation. Second, the current study did not include positive controls, which makes it difficult to benchmark DD’s anti-fibrotic efficacy. Although Western blotting data showed that DD decreased the protein expression of HIF-1α and TGF-β/Smad signaling pathways, the upstream signaling pathways potentially related to hypoxia, such as PI3K/Akt and MAPK, were not validated. Third, rutin decreased the protein expressions of HIF-1α, TGF-β, p-Smads/Smads, and the application of the inhibitor of HIF-1a, PX-478, supported that HIF-1α was possibly involved in the antifibrotic effects of rutin in HK-2 cells; however, further mechanical experiments, such as HIF-1α knockdown/overexpression, Smad reporter assays, TGF-β1 stimulation with pathway inhibitor comparison, nuclear translocation assays, or direct binding assays for rutin, such as CETSA or SPR, are needed to provide more evidence for the inhibitory mechanism. In addition, the amount of rutin in DD, the in vivo pharmacokinetic/tissue exposure and its contribution to the effect of DD were not directly examined. Therefore, further experiments are required to clarify the anti-fibrosis role of rutin in DD. In all, this study suggests that DD exerts a reno-protective effect in AA I-induced renal fibrosis, which may be associated with the inhibition of HIF-1α and TGF-β/Smad signaling pathways. In vitro experiments indicate that rutin may be a potential bioactive constituent that potentially targets HIF-1α signaling pathways.

## 4. Materials and Methods

### 4.1. Materials

Serum biochemistry reagents, including aspartate aminotransferase (AST), creatinine (CRE), alanine aminotransferase (ALT), blood urea nitrogen (BUN), alkaline phosphatase activity (ALP), and total bilirubin (TBIL-1), were obtained from Hua Sin Science Co., Ltd. (Guangzhou, China). Reagents for hematology analysis were bought from Siemens Healthcare GmbH (Erlangen, Germany). The hematoxylin–eosin (H&E) and Masson trichrome staining kits were purchased from Servicebio (Beijing, China). All primers were obtained from Sangon Biotech (Shanghai) Co., Ltd. (Shanghai, China). The antibodies of HIF-1α, PHD2, PHD4, TGF-β, and GAPDH were obtained from Abcam (Cambridge, UK). The Smad 2/3 and p-Smad 2/3 antibodies were purchased from Cell Signaling Technology (Danvers, MA, USA). Acetonitrile, formic acid, and ammonium acetate were of LC-MS grade and obtained from Fisher Scientific (Waltham, MA, USA). The Collagen I and fibronectin antibodies were obtained from Invitrogen (Shanghai, China). The EPO antibody was purchased from Proteintech Group, Inc. (Wuhan, China). Aristolochic acid I (AA I) (purity > 98%), chlorogenic acid (purity, 96.3%), and quercetin (purity, 98.7%) were purchased from the National Institutes for Food and Drug Control (Beijing, China). Rutin (purity ≥ 95%) and ononin (purity ≥ 97.0%) were purchased from Aladin Scientific Corp. (Shanghai, China). Isorhamnetin (purity ≥ 98.0%), calycosin-7-O-glucoside (purity ≥ 98.0%), and the BCA protein assay kit were obtained from Beyotime Biotechnology (Shanghai, China). PX-478 was purchased from MedChemExpress LLC (Shanghai, China).

### 4.2. Network Pharmacology

#### 4.2.1. “Drug–Disease” Intersection Targets

The chemical constituents in DD were collected via UPLC-MS/MS detection and from the Traditional Chinese Medicine System Pharmacology Database and Analysis Platform (TCMSP, https://tcmsp-e.com/tcmsp.php, accessed on 8 October 2025) and published studies. Among these, constituents with oral bioavailability (OB) ≥ 30% and CLaSP score ≥ 0.9 were chosen for further analysis. The protein targets of chemical constituents from the TCMSP platform and BATMAN (target prediction threshold ≥ 0.9) were converted into gene symbols using UniProt (https://www.uniprot.org/, accessed on 9 October 2025).

Renal fibrosis-related genes were screened with a minimum disease gene score ≥ 0.2, using the Human Gene Database (GeneCards, https://www.genecards.org, accessed on 11 October 2025) and the Therapeutic Target Database (TTD, http://db.idrblab.net/ttd/, accessed on 12 October 2025), with all duplicate entries removed. A “drug–disease” intersectional target database was constructed by combining the disease targets and the drug targets. Additionally, the common targets were visualized using a Venn diagram (https://bioinformatics.com.cn, accessed on 12 October 2025).

#### 4.2.2. Protein–Protein Interaction (PPI) Network and “Components–Targets-Disease” Network

The common targets obtained from the Venn diagram were uploaded to the STRING database (https://string-db.org/, accessed on 18 October 2025) to analyze their interactions. Subsequently, the PPI network (minimum PPI confidence ≥ 700) was obtained using Cytoscape 3.8.0 (https://cytoscape.org, accessed on 18 October 2025).

The “components–targets-disease” network for DD was obtained from Cytoscape 3.8.0 to elucidate its pharmacological mechanisms of action. The significance of each node within the network was determined by the degree value, with higher degree values indicating greater importance. The core active constituents were identified based on their degree values.

#### 4.2.3. Gene Ontology and Kyoto Encyclopedia of Genes and Genomes Enrichment Analyses

The core targets were imported into the Metascape database. Gene Ontology (GO) and Kyoto Encyclopedia of Genes and Genomes (KEGG) enrichment analyses were conducted based on the core targets (enrichment analysis FDR < 0.05, enrichment analysis *p*-value < 0.05). Biological process (BP), molecular function (MF), and cellular component (CC) were included in GO enrichment analysis. Parameters were set as follows: minimum overlap, 3; *p*-value cutoff, 0.01; minimum enrichment threshold, 1.5.

### 4.3. Drug Preparation

*Astragalus membranaceus* (Fisch.) Bunge (AM) and *Angelica sinensis* (AS) were obtained from Tongren herbal pharmacy (Beijing, China). DD was obtained with AM/AS at a ratio of 5:1. To prepare the extract of AM, AS, and DD, herbal materials were first soaked with 10 times distilled water for 30 min. Next, decoctions were obtained by reflux twice at 100 °C for 1 h. The filtrate was collected and frozen at −80 °C until further use. Then, the centrifuge was pre-cooled to 4 °C. DD was centrifuged at a speed of 12,000 rpm for 15 min. Finally, the suspension was determined using UPLC-MS.

### 4.4. Chromatographic Separation and Mass Spectrum Conditions

The active constituents of DD were determined using a Waters I-Class UPLC system (Waters Corporation, Milford, CT, USA). A Waters BEH C18 column (2.1 × 100 mm, 1.7 μm) was applied to separate the constituents at 35 °C. A 5 μL sample was injected into the UPLC-MS system. Separations were obtained from gradient elution (0–2.0 min, 10% B; 2.0–21.0 min, 10–95% B; 21.0–26.0 min, 95% B; 26.0–26.5 min, 95–10% B; 26.5–30.0 min, 10% B) with water and acetonitrile (both containing 0.1% formic acid) as mobile phases. The flow rate was 0.3 mL/min.

The active constituents were detected by Waters Xevo G2-XS Q/Tof mass spectrometry (Waters Corporation, Milford, CT, USA) with a capillary voltage of 3.5 kV, source temperature 120 °C, cone voltage 40 V, and desolvation temperature 500 °C. Data was acquired by MS^E^ scan with a scan range of *m*/*z* 50–1000 in both positive and negative modes. A collision energy of 10–50 V was used to obtain precursor ions and fragment ions.

### 4.5. Animals and Treatment

C57BL/6J mice at 20–22 g were obtained from Beijing Weitonglihua Laboratory Animal Technology Co. Ltd. (Beijing, China). Mice were housed under standard conditions and allowed a 7-day acclimatization prior to administration. Then, 80 mice were divided into 8 groups randomly, with 10 mice in each group. The renal fibrosis mouse model was acquired by intraperitoneal injection of AA I at 3.0 mg/kg every other day for two consecutive weeks. Mice in the control group were administered water. Animals in the treatment groups were then given AM (4.5 g/kg, 9.0 g/kg), AS (0.9 g/kg, 1.8 g/kg), and DD (4.5 g/kg, 9.0 g/kg) at two doses by oral gavage for 4 weeks. The low-dose DD is the clinically equivalent dose for humans. The animal weights were recorded twice a week. Before sacrifice, mice were fasted for 12 h. Blood samples were obtained and centrifuged for 15 min at 3500 rpm to obtain serum.

### 4.6. Hematology and Serum Biochemical Assays

The whole blood was anticoagulated with 10% EDTA-K_2_·2H_2_O (1:49) before hematology analysis, and the test was carried out utilizing an automatic hematology analyzer (SIEMENS ADVIA2120, Siemens Healthcare Diagnostics Inc., Tarrytown, NY, USA). The non-anticoagulated serum was applied for biochemical determination with an automatic biochemical analyzer (TOSHIBA 40FR, Toshiba Corporation, Tokyo, Japan), including BUN, AST, Cre, ALT, TBIL, and ALP.

### 4.7. Histopathology

Renal tissue samples were first fixed with 4% paraformaldehyde solution. Then, the tissues were embedded in paraffin and cut into 4 µm thick pieces. The slices were subsequently stained with H&E and Masson trichrome staining kits, respectively. Histological images were examined using Olympus microscope BX63 (Olympus Corporation, Tokyo, Japan) at a magnification of 400×.

### 4.8. Real-Time Quantitative PCR Analysis

The primer sequences for qRT-PCR analysis were listed in Table 1. Total RNA from renal tissues was isolated with a RNeasy Total RNA Isolation Kit (TaKaRa Bio Inc., Kusatsu, Japan) and reverse-transcribed into cDNA. SYBR Green quantitative PCR analysis reactions were performed using the Roche 480 II detection system (Roche, Basel, Switzerland). Each reaction consisted of 10 μL of SYBR Green, 4.0 μL of cDNA, 0.8 μL of each primer pair (10 mmol/mL), and 5.2 μL of distilled water. The GAPDH gene was simultaneously detected as the endogenous control. Relative gene expression levels were quantified by means of the 2^−ΔΔCt^ method, and the results are shown as the fold change relative to the control or the model group.

### 4.9. Western Blotting

Proteins from renal tissues in mice or HK-2 cells were extracted with RIPA lysis buffer and quantified using the BCA method. After separation on a 12% SDS-PAGE gel, proteins were transferred to PVDF membranes to facilitate subsequent incubation with antibodies. The membranes were blocked with 5% defatted milk for TGF-β1, GAPDH, HIF-1α, collagen I, fibronectin and Smad 2/3 or 5% BSA for *p*-Smad 2/3 for 1 h, and then subsequently incubated with diluted primary antibodies at 4 °C overnight as follows: TGF-β1 (1:1000), GAPDH (1:5000), HIF-1α (1:1000), collagen I (1:2000), fibronectin (1:1000), Smad 2/3 (1:1000) and *p*-Smad 2/3 (1:1000). After incubation with secondary antibodies and luminol, the protein bands were visualized with ChemiScope 6050 (Clinx, Shanghai, China) and analyzed using Image J (version 1.54), respectively.

### 4.10. Cell Culture and Treatment

HK-2 cells were cultured in DMEM/F12 medium containing 10% fetal bovine serum, 100 U/mL penicillin, and 100 μg/mL streptomycin at 37 °C in a humidified incubator with 5% CO_2_. Overall, 5000 HK-2 cells were seeded in each well of 96-well plates overnight. Next, the cells were incubated with 6 test drugs for 24 and 48 h at various concentrations (3.125, 6.25, 12.5, 25, 50, 100, 200 μM for AA I; 1.563, 3.125, 6.25, 12.5, 25, 50, 100 μM for rutin; 3.125, 6.25, 12.5, 25, 50, 100, 200 μM for isorhamnetin, quercetin, calycosin-7-O-glucoside, ononin, and chlorogenic acid). Finally, the viabilities of HK-2 cells were assessed according to the instructions of the CCK8 kit.

HK-2 cells were incubated with AA I for 48 h to induce renal fibrosis. Cells were divided into eight groups: (1) control, (2) AA I (10 μM), (3) AA I (10 μM) + isorhamnetin (10 μM), (4) AA I (10 μM) + quercetin (10 μM), (5) AA I (10 μM) + rutin (10 μM), (6) AA I (10 μM) + ononin (10 μM), (7) AA I (10 μM) + chlorogenic acid (10 μM), and (8) AA I (10 μM) + calycosin-7-O-glucoside (10 μM). The supernatant and cell lysate in different groups were used to determine the expression of HIF-1α, TGF-β, and collagen I by ELISA. The protein expressions of HIF-1α, TGF-β, Smad 2/3, p-Smad 2/3, collagen I, and GAPDH in rutin (5, 10, 20 μM)-, PX-478 (20 μM)-, and rutin (20 μM) + PX-478 (20 μM)-treated HK-2 cells were further determined by Western blot analysis.

### 4.11. Molecular Docking

The protein structures of factor inhibiting HIF-1α (3KCY, 2.59 Å) and TGF-beta receptor I kinase (1PY5, 2.30 Å) were obtained from the RCSB protein database (RCSB PDB, https://www.wwpdb.org/, accessed on 12 December 2025) [58,59]. Modifications and ligands were separated from the crystals, and water molecules were removed using PyMOL software (version 2.4.0). The 3D structures of test drugs were obtained from PubChem. AutoDock Vina (version 1.2.5) was applied to predict the binding affinities of test drugs to HIF-1α and TGF-β. The receptor–ligand pairs were ranked based on their minimum binding energies, with the highest-ranking pair extracted and organized using a batch script. The receptor–ligand with the minimum binding energy was regarded as the optimal combination of the test drug and protein. Additionally, the molecular docking images were obtained from PyMOL software.

### 4.12. Statistical Analysis

Data were represented as the mean ± standard deviation (SD). One-way analysis of variance (ANOVA) and the least significant difference (LSD) test were used to evaluate the significant differences between differently treated groups (*p* < 0.05).

## 5. Conclusions

This study integrates chemical profiling, network pharmacology, molecular docking, and in vitro and in vivo validations to explore the potential protective effects of DD against AA I-induced renal fibrosis. DD improves renal functions and attenuates AA I-induced renal fibrosis. The protective effect is potentially related to HIF-1α and TGF-β/Smad signaling pathways. In addition, molecular docking and in vitro assays indicated that rutin may be a candidate bioactive component in DD, and its potential target might be HIF-1α. However, further studies concerning the amount of rutin in DD, the in vivo exposure, and its contribution to the effect of DD were still necessary.

## Figures and Tables

**Table 1:** Primer sequences.

mRNA	Primers
TGF-β	forward—CAACAATTCCTGGCGTTACCTTGG reverse—TGTATTCCGTCTCCTTGGTTCAGC
Smad 2	forward—TCGTCCATCTTGCCATTCACTCC reverse—CCATTCTGCTCTCCACCACCTG
HIF-1α	forward—TGCCACTGCCACCACAACTG reverse—TGCCACTGTATGCTGATGCCTTAG
PHD4	forward—CCAGACCAGGCGATAGTGAAGAC reverse—ACACCATCAGCACCAAGAAGTAGG
Collagen I	forward—ACAGGCGAACAAGGTGACAGAG reverse—AGGAGAACCAGGAGAACCAGGAG
Fibronectin	forward—CACCGACGAAGAGCCCTTACAG reverse—CCTTGTGCCTCCTCTGGTTCTG
GADPH	forward—GGTTGTCTCCTGCGACTTCA reverse—TGGTCCAGGGTTTCTTACTCC

## Data Availability

The original contributions presented in this study are included in the article and Supplementary Materials. Further inquiries can be directed to the corresponding authors.

## Supplementary Materials

The following supporting information can be downloaded at: https://www.mdpi.com/article/10.3390/ph19081206/s1, Table S1: Chemical constituents in DD determined using UPLC-MS/MS; Table S2: Top 10 targets with degree value; Table S3: Top 10 components with degree value; Table S4: Docking scores of 17 active components with HIF-1α and TGF-β; Figure S1: Level changes in serum biochemical indexes of mice administered with AML, AMH, ASL, ASH, DDL, and DDH for 4 weeks. (A) AST, (B) ALT, (C) ALP, (D) TBIL-1. Significantly different from the control group (***, *p* < 0.001; ****, *p* < 0.0001, *n* = 8). Significantly different from the AA I group (^##^, *p* < 0.01; ^####^ *p* < 0.0001, *n* = 8); Figure S2: DD relieved AA I induced anemia. (A) Simplified diagram of mice administrated with test drugs. (B–G) Hematology of mice in control, AA I, AML, AMH, ASL, ASH, DDL, and DDH group, respectively. RBC (10^6^ cells/μL), HGB (g/dL), HCT (%), MCV (fL), MCH (pg), and RDW (%). (H,I) The protein expression of EPO in control, AA I, AML, AMH, ASL, ASH, DDL and DDH groups, respectively. Statistical comparisons: ***, *p* < 0.001; ****, *p* < 0.0001 vs. control. ^#^, *p* < 0.05; ^##^, *p* < 0.01; ^###^, *p* < 0.001; ^####^, *p* < 0.0001 vs. model (*n* = 8 for hematology and *n* = 3 for western blotting). Figure S3. Hematology of mice in control, AA I, AML, AMH, ASL, ASH, DDL, and DDH group, respectively. WBC (10^6^ cells/μL), LYMPH (%), NEUT (%), MONO (%), BASO (%), and MPV (fL), PLT (10^6^ cells/μL), PCT (%), and PDW (%). Significantly different from the control group (**, *p* < 0.01; ***, *p* < 0.001; *n* = 8). Significantly different from AA I group (^#^, *p* < 0.05; ^##^, *p* < 0.01; ^###^, *p* < 0.001; *n* = 8).

## Abbreviations

The following abbreviations are used in this manuscript:

CKD	Chronic kidney disease
EMT	Epithelial-to-mesenchymal transition
ECM	Extracellular matrix
ESRD	End-stage renal failure
TGF-β	Transforming growth factor-β
TEs	Renal tubular epithelial cells
HIF-1α	Hypoxia-inducible factor-1α
PHD	HIF-1α-associated prolyl hydroxylase
TCM	Traditional Chinese medicine
DD	Dangguibuxue decoction
AS	*Angelica sinensis* (Oliv.) Diels
AM	*Astragalus membranaceus* (Fisch.) Bge.
EPO	Erythropoietin
NF-κB	Nuclear factor kappa-B
GATA2	Recombinant GATA binding protein 2
AST	Aspartate aminotransferase
ALT	Alanine aminotransferase
ALP	Alkaline phosphatase activities
Cre	Creatinine
BUN	Blood urea nitrogen
TBIL-1	Total bilirubin
H&E	Hematoxylin-eosin
TCMSP	Traditional Chinese medicine system pharmacology database and analysis platform
OB	Oral bioavailability
TTD	Therapeutic target database
PPI	Protein–protein interaction
GO	Gene ontology
KEGG	Kyoto Encyclopedia of Genes and Genomes
BP	Biological process
MF	Molecular function
CC	Cellular component
UPLC	Ultra-performance liquid chromatography
RBC	Red blood cell
WBC	White blood cell
HGB	Hemoglobin
HCT	Hematocrit
PLT	Blood platelet number
MCV	Mean corpuscular volume
MCH	Mean corpuscular hemoglobin
MCHC	Mean corpuscular hemoglobin concentration
PCT	Platelet crit
PDW	Platelet distribution width
LYMPH	Lymphocyte
NEUT	Neutrophil count
BASO	Basophil
MONO	Monocyte
MPV	Mean platelet volume
AA I	Aristolochic acid I
AAN	Aristolochic acid nephropathy
CCK8	Cell counting kit 8
ELISA	Enzyme-linked immunosorbent assay
