# Supplementary material for: Dangguibuxue Decoction Attenuated AA I-Induced Renal Fibrosis: Integrating Network Pharmacology and Experimental Validation
Source: Pharmaceuticals (Basel). 2026 Aug 1;19(8):1206. doi: 10.3390/ph19081206 (PMC13516570; doi:10.3390/ph19081206)
Supplement: Supplementary file 1 [file pharmaceuticals-19-01206-s001.zip › pharmaceuticals-4348495-supplementary.pdf]

Supplementary Information

1. Chemical profile of Dangguibuxue decoction (DD) by UPLC-MS/MS

Table S1. Chemical constituents in DD determined using UPLC-MS/MS.

No.	Component	Formula	RT (min)	Molecular weight	Observed m/z	Adducts	Mass error (ppm)	MS^E Fragments
1	γ-aminobutyric acid	$C_4H_9NO_2$	0.77	103.0633	104.0704	$[M+H]^+$	-2.1	86.0595
2	L-Asparagine	$C_4H_8N_2O_3$	0.77	132.0535	133.0605	$[M+H]^+$	-2.3	116.0333, 90.0545, 88.0392, 87.0545, 85.0281
3	betaine	$C_5H_{11}NO_2$	0.79	117.0790	118.0856	$[M+H]^+$	-5.8	-
4	glusose	$C_6H_{12}O_6$	0.89	180.0634	203.0523	$[M+Na]^+$	-1.5	163.0591, 145.0499, 132.0767, 127.0385, 112.0503, 85.0283
5	nicotinic acid	$C_6H_5NO_2$	0.95	123.0320	124.0391	$[M+H]^+$	-1.5	80.0487
6	adenine	$C_5H_5N_5$	1.15	135.0545	136.0612	$[M+H]^+$	-0.2	119.0348
7	prolinum	$C_5H_9NO_2$	1.23	115.0633	116.0701	$[M+H]^+$	-4	100.0754, 85.0278
8	L-Arginin	$C_6H_{14}N_4O_2$	1.25	174.1117	175.1185	$[M+H]^+$	-0.3	114.0542
9	heriguard	$C_{16}H_{18}O_9$	2.4	354.0951	355.1022	$[M+H]^+$	-2.3	163.0382, 145.0281, 135.0426
10	isoflavanone	$C_{17}H_{16}O_6$	3.08	316.0947	315.0870	$[M-H]^-$	-1.3	299.0512
11	vanillic acid	$C_8H_8O_4$	3.36	168.0423	169.0495	$[M+H]^+$	-1.7	151.0386, 137.0600, 135.0445, 107.0484, 93.0336
12	flavaxin	$C_{17}H_{20}N_4O_6$	3.89	376.1383	377.1440	$[M+H]^+$	-3.1	243.0868, 236.1491, 167.0334
13	quercetin	$C_{15}H_{10}O_7$	3.92	463.1026	462.0873	$[M-H]^-$	-4.2	199.0265, 247.0612, 115.0987, 429.1638, 205.0934

14	kaempferol	$C_{15}H_{10}O_6$	3.99	286.0477	331.0444	$[M+HCOO]^-$	-4.8	191.0417
15	daidzein	$C_{15}H_{10}O_4$	4.49	254.0579	255.0650	$[M+H]^+$	-0.6	131.0495
16	(2S)-2-amino-4-guanidinooxybutanoic acid	$C_5H_{12}N_4O_3$	4.52	176.0909	175.0831	$[M-H]^-$	-3.0	159.0869, 118.0732, 117.0419, 157.0730, 131.0931, 129.0780
17	calycosin-7-O-beta-glucoside	$C_{22}H_{22}O_{10}$	5.21	446.1213	447.1280	$[M+H]^+$	-1.2	270.0519, 413.0820, 253.0494, 137.0232
18	calycosin	$C_{16}H_{12}O_5$	5.21	284.0684	285.0757	$[M+H]^+$	-0.3	270.0519, 137.0232, 253.0494
19	isoliquiritigenin	C15H12O4	5.21	256.0736	257.0800	$[M+H]^+$	-3.8	137.0232, 131.0486, 105.0328, 121.0645
20	ferulic acid	$C_{10}H_{10}O_4$	5.25	194.0579	195.0647	$[M+H]^+$	-2.6	177.0542, 149.0597, 145.0281, 134.0360
21	7-O-methylisomucronulatol	$C_{18}H_{20}O_5$	5.34	316.1311	375.1439	$[M+CH_3COO]^-$	-2.6	212.1028, 337.1082
22	7,2'-dihydroxy-3',4'-dimethoxyisoflavone-7-O-β-D-glucoside	$C_{23}H_{24}O_{11}$	5.35	476.1319	477.1383	$[M+H]^+$	-1.8	413.1198, 355.1165, 137.0590
23	lariciresinol	$C_{20}H_{24}O_6$	5.77	360.1573	359.1491	$[M-H]^-$	-3	283.1004, 300.1731, 281.0856
24	alpha-L-rhamnose	$C_6H_{12}O_5$	5.91	164.0684	163.0621	$[M-H]^-$	-0.6	149..0824, 119.0724
25	isomucronulatol-7,2'-di-O-glucosiole	$C_{29}H_{38}O_{15}$	6.06	626.2211	644.2533	$[M+NH_4]^+$	-2.5	401.1584, 429.1531, 383.1494, 123.0399
26	Fucopyranose, L	$C_6H_{12}O_5$	6.08	164.0685	223.0815	$[M+CH_3COO]^-$	-3.5	149.0458, 135.0669, 121.0514, 119.0725
27	calycosin	$C_{16}H_{12}O_5$	6.18	284.0685	285.0753	$[M+H]^+$	-0.3	270.0531, 269.0448, 253.0485, 137.0234, 134.0372
28	rhamnocitrin-3-O-glucoside	$C_{22}H_{22}O_{11}$	6.18	462.1162	463.1231	$[M+H]^+$	-0.9	270.0517, 253.0485, 137.0227, 245.0807
29	nonanedioic acid	$C_9H_{16}O_4$	6.41	188.1049	211.0933	$[M+Na]^+$	-3.8	125.0948

30	rutin	$C_{27}H_{30}O_{16}$	6.49	610.1534	609.1434	$[M-H]^-$	-5	529.0960, 289.0936, 275.1130
31	coniferyl ferulate	$C_{20}H_{20}O_6$	6.61	356.1260	357.1312	$[M+H]^+$	-0.6	343.1165, 137.0596, 131.0487
					355.1168	$[M-H]^-$	-2.7	341.1045, 321.0768, 307.0816, 299.0954, 137.0471
32	calycosin	$C_{16}H_{12}O_5$	6.82	284.0685	285.0753	$[M+H]^+$	-0.1	270.0521, 253.0492, 137.0226, 134.0353
33	E-6,7-dihydroxydihydroligustilide	$C_{12}H_{16}O_4$	6.93	224.1049	247.0939	$[M+Na]^+$	-0.9	207.1017, 189.0910, 161.0957, 179.1068, 167.0701
34	senkyunolide I	$C_{12}H_{16}O_4$	6.93	224.1049	247.0934	$[M+Na]^+$	-1.2	207.1013,191.0710, 189.0901, 179.1075, 175.0733
35	senkyunolide F	$C_{12}H_{14}O_3$	6.94	206.0943	207.1012	$[M+H]^+$	-1.9	189.0901, 191.0710, 179.1075, 175.0733
36	2-valerylbenzoic acid	$C_{12}H_{14}O_3$	6.94	206.0943	207.1012	$[M+H]^+$	-4.3	189.0901, 161.0951, 147.0777, 123.0430
37	ononin	$C_{22}H_{22}O_9$	7.04	430.1264	431.1335	$[M+H]^+$	-0.3	253.0489, 207.0635, 145.0292, 136.0145
38	formononetin	$C_{16}H_{12}O_4$	7.04	268.0736	269.0808	$[M+H]^+$	-0.1	237.0543, 137.0225, 136.0145
39	Astrapterocarpan-3-O-glucoside	$C_{23}H_{26}O_{10}$	7.5	462.1526	463.1552	$[M+H]^+$	-0.7	286.0840, 251.0573, 167.0699, 151.0346
40	(6aR,11aR)-9,10-dimethoxy-6a,11a-dihydro-6H-benzofurano[3,2-c] chromen-3-ol	$C_{17}H_{16}O_5$	7.51	300.0998	301.1065	$[M+H]^+$	-1.7	271.0920, 286.0839, 285.0754, 167.0699, 151.0346,147.0435, 123.0402
41	isomucronulatol	$C_{17}H_{18}O_5$	7.73	302.1154	303.1220	$[M+H]^+$	-1.8	193.0849, 181.0855, 167.1059,149.0585, 123.0439
42	astraisoflavanin	$C_{23}H_{28}O_{10}$	7.73	464.1683	465.1750	$[M+H]^+$	-0.3	429.1510, 411.1442, 329.2064,

								167.0701
43	rhamnocitrin	$C_{16}H_{12}O_6$	7.78	300.0634	301.0699	$[M+H]^+$	-0.2	269.0824, 254.0582, 108.0218, 163.0410, 134.0365
44	5'-hydroxyisomuronulatol-2',5'-di-O-glucoside	$C_{29}H_{38}O_{16}$	8.2	642.2160	701.2277	$[M+CH_3COO]^-$	-3.0	595.1987, 511.1424, 505.1747, 477.1439, 404.1146, 387.1271,135.0686
45	E-butylidenephthalide	$C_{12}H_{12}O_2$	8.48	188.0837	189.0902	$[M+H]^+$	-4.5	161.0945, 143.0850, 128.0618, 115.0539
46	scopoletol	$C_{10}H_8O_4$	9.3	192.0423	193.0494	$[M+H]^+$	-0.9	
47	sedanolide	$C_{12}H_{18}O_2$	9.45	194.1307	195.1373	$[M+H]^+$	-3.4	181.1206, 167.1421, 163.1105, 139.1110
48	isoamylbenzene	$C_{11}H_{16}$	9.45	148.1252	149.1320	$[M+H]^+$	-3.1	107.0847, 105.0697, 93.0681, 91.0537, 133.1010
49	vanillin	$C_8H_8O_3$	9.5	152.0473	170.0806	$[M+NH_4]^+$	-1.7	137.0604
50	coumarin	$C_9H_6O_2$	9.78	146.0368	147.0439	$[M+H]^+$	-4.7	131.0480, 103.0538, 95.0488
51	astragaloside IV	$C_{41}H_{68}O_{14}$	9.78	784.4609	807.4492	$[M+Na]^+$	-1.1	587.3938, 569.3822, 473.3610, 437.3407, 455.3507
52	asernestioside B	$C_{49}H_{80}O_{19}$	9.8	972.5294	971.5177	$[M-H]^-$	-4.5	873.4503, 487.2711, 343.2273, 341.1251, 327.2345
53	astragaloside III	$C_{41}H_{68}O_{14}$	9.93	784.4609	807.4500	$[M+Na]^+$	-0.2	473.3624, 455.3506, 437.3408, 419.3297
54	isotetandrine	$C_{38}H_{42}N_2O_6$	10.07	622.3043	681.3156	$[M+CH_3COO]^-$	-0.5	525.2309，503.2531, 401.0992，375.1980, 209.1016
55	soyasaponin I	$C_{48}H_{78}O_{18}$	10.8	942.5188	943.5250	$[M+H]^+$	-1.1	781.4726, 797.4667, 605.4417, 599.3987, 423.3614
56	senkyunolide-D	$C_{12}H_{14}O_4$	11.19	222.0892	245.0773	$[M+Na]^+$	-4.4	149.0229, 121.0295

57	palmitic acid	$C_{16}H_{32}O_2$	11.63	256.2402	274.2735	$[M+NH_4]^+$	-2.0	124.0869, 228.2070, 198.1589, 196.1824
58	astragaloside II	$C_{43}H_{70}O_{15}$	11.76	826.4369	871.4715	$[M+COOH]^+$	-3.1	825.4298, 651.4241, 179.0245,
59	astragaloside I	$C_{45}H_{72}O_{16}$	11.95	868.4820	891.4712	$[M+Na]^+$	-0.1	689.4257, 671.4149, 455.3517, 437.3409, 419.3299
60	safrol	$C_{10}H_{10}O_2$	12.11	162.0681	163.0751	$[M+H]^+$	-1.4	145.0642, 135.0800, 95.0495
61	3,4-dimethylbenzaldehyde	$C_9H_{10}O$	12.11	134.0732	135.0802	$[M+H]^+$	-1.9	107.0851, 91.0537
62	senkyunolide K	$C_{12}H_{16}O_3$	12.11	208.1099	209.1171	$[M+H]^+$	-0.6	177.0906, 119.0848, 107.0851, 103.0535
63	2-Methylhexadecanoic acid	$C_{17}H_{34}O_2$	12.26	270.2559	288.2892	$[M+NH_4]^+$	-1.9	271.1548, 183.1738
64	isomer of ligustilide	$C_{12}H_{14}O_2$	12.31	190.0994	191.1065	$[M+H]^+$	-0.9	177.0899, 167.1070, 107.0851, 91.0537
65	13-hydroxy-9,11-octadecadienoic acid	$C_{18}H_{32}O_3$	12.34	296.2351	297.2418	$[M+H]^+$	-1.8	209.1529, 177.1269, 119.0852, 91.0535
66	linolenic acid	$C_{18}H_{30}O_2$	12.34	278.2246	279.2309	$[M+H]^+$	-3.8	209.1529, 177.1269, 119.0852, 91.0535, 107.0850
67	isomer of astragaloside I	$C_{45}H_{72}O_{16}$	12.46	868.4820	891.4712	$[M+Na]^+$	-0.72	671.4150, 411.1986, 455.3473, 357.1901, 237.1489
68	linoleic acid	$C_{18}H_{32}O_2$	12.52	280.2402	298.2735	$[M+NH_4]^+$	-1.7	263.2365, 191.1425, 111.0795, 97.1016
69	butylphthalide	$C_{12}H_{14}O_2$	12.93	190.2380	191.106	$[M+H]^+$	-4.5	163.1104,145.1005
70	senkyunolide A	$C_{12}H_{16}O_2$	13.06	192.1150	193.1215	$[M+H]^+$	-3.9	163.1116, 123.1153, 147.1148, 81.0693, 109.0998
71	Z-ligustilide	$C_{12}H_{14}O_2$	13.44	190.0994	191.1066	$[M+H]^+$	-3.3	149.0581, 145.1014, 135.0437
72	6,7,3',8'-diligustilide	$C_{24}H_{28}O_4$	13.44	380.1988	381.2049	$[M+H]^+$	-2.9	307.1674, 263.1268, 277.1426
73	(E)-octadec-3-ene	$C_{18}H_{36}$	13.70	252.2817	270.3148	$[M+NH_4]^+$	-2.8	85.101

74	isomer of butylphthalide	$C_{12}H_{14}O_2$	14.19	190.2380	191.1060	$[M+H]^+$	-3.1	145.1002, 135.1167
75	pentadeca-7,10-diynoic acid	$C_{15}H_{22}O_2$	14.78	234.1620	235.1686	$[M+H]^+$	-2.7	187.1115, 179.1064, 117.0699, 105.0694, 89.0390
76	senkyunolide C	$C_{12}H_{12}O_3$	16.13	204.0786	205.0852	$[M+H]^+$	-2.9	149.0230, 121.0285, 93.0326
77	docosanoate	$C_{22}H_{44}O_2$	17.5	340.3341	358.3671	$[M+NH_4]^+$	-2.4	85.1007
78	lecithin	$C_{36}H_{72}NO_8P$	22.3	677.4996	722.4959	$[M+HCOO]^-$	-0.2	341.1241, 461.3677
79	acetylastragaloside I	$C_{47}H_{74}O_{17}$	24	910.4926	955.4574	$[M+HCOO]^-$	-4.7	825.4103, 739.4070, 549.2189, 512.3485, 371.2569

2. Network pharmacology

Table S2. Top 10 targets with degree value.

Name	Degree
TNF	33
INS	30
PTGS2	29
PPARG	29
TP53	26
HIF1A	25
STAT3	25
REN	23
PPARA	23
TGFB1	23

Table S3. Top 10 components with degree value.

Compounds	Degree
Quercetin	138
Kaempferol	57
Formononetin	34
Isorhamnetin	31
Rutin	23
Nicotinic acid	21
Rhamnocitrin	19
Linolenic acid	16
Daidzein	12
Caffeate	12

3 DD attenuates AA I-induced renal fibrosis in mice

3.1 Serum biochemistry test

The serum AST, ALP, and TBIL-1 in mice of AA I group had a significant increase compared with that of control group ($P < 0.001$, or $P < 0.0001$). There was no significant difference in AST, ALP, and TBIL between treated groups and AA I group (**Figure S1**). The serum ALT in ASL, ASH, DDL, and DDH groups were significantly increased ($P < 0.01$ or $P < 0.0001$).

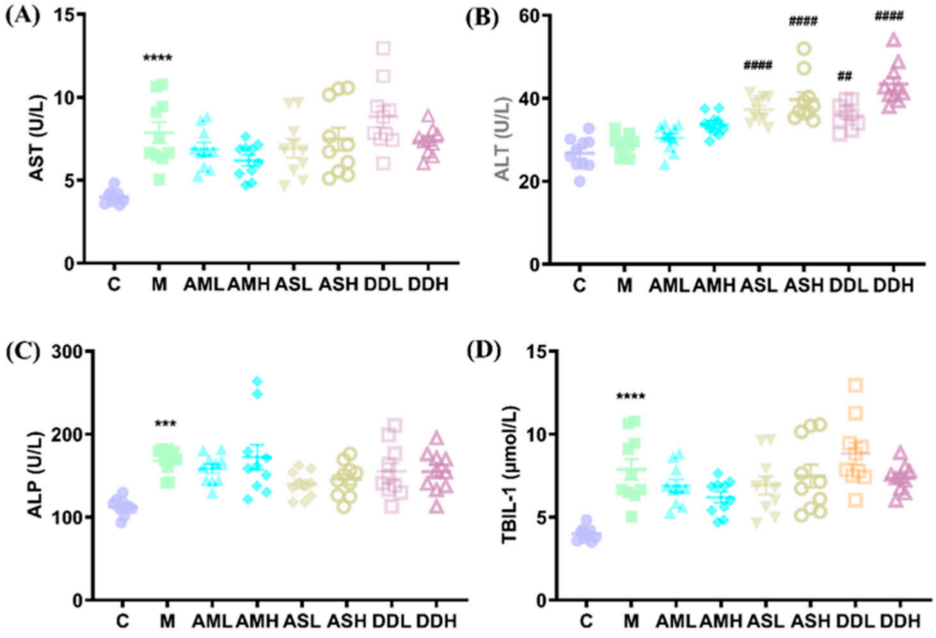

Figure S1. Level changes in serum biochemical indexes of mice administered with AML, AMH, ASL, ASH, DDL, and DDH for 4 weeks. (A) AST, (B) ALT, (C) ALP, (D) TBIL-1. Significantly different from the control group (***, $P < 0.001$; ****, $P < 0.0001$, n=8). Significantly different from the AA I group ($^{\#\#}$, $P < 0.01$; $^{\#\#\#\#}$ $P < 0.0001$, n=8).

3.2 Hematology

Mice were treated according to the scheme shown in the Figure S2A. After treatment with AA I, the RBC, HGB, HCT, MCV, and RDW values in mice were significantly decreased ($P < 0.0001$), suggesting the occurrence of anemia (Figures S2B-G). AS and DD are usually used to replenishing blood in clinical treatment. Therefore, hematology parameters were compared to explore that whether they could relive AA I induced anemia. As shown in Figures S2B-G, the HGB and HCT in AML treated mice had a significant increment ($P < 0.05$). ASH greatly increased the RBC, HGB, HCT, and MCV ($P < 0.05$, $P < 0.01$, $P < 0.001$). DDL and DDH increased the RBC, HGB, HCT, MCV, and RDW remarkably ($P < 0.05$, $P < 0.01$, $P < 0.001$, or $P <$ 0.0001). In addition, the protein expression of EPO was evidently upregulated in AMH, ASH, DDL, and DDH groups ($P < 0.05$, $P < 0.01$, or $P < 0.001$) (Figures S2H-I**)**.

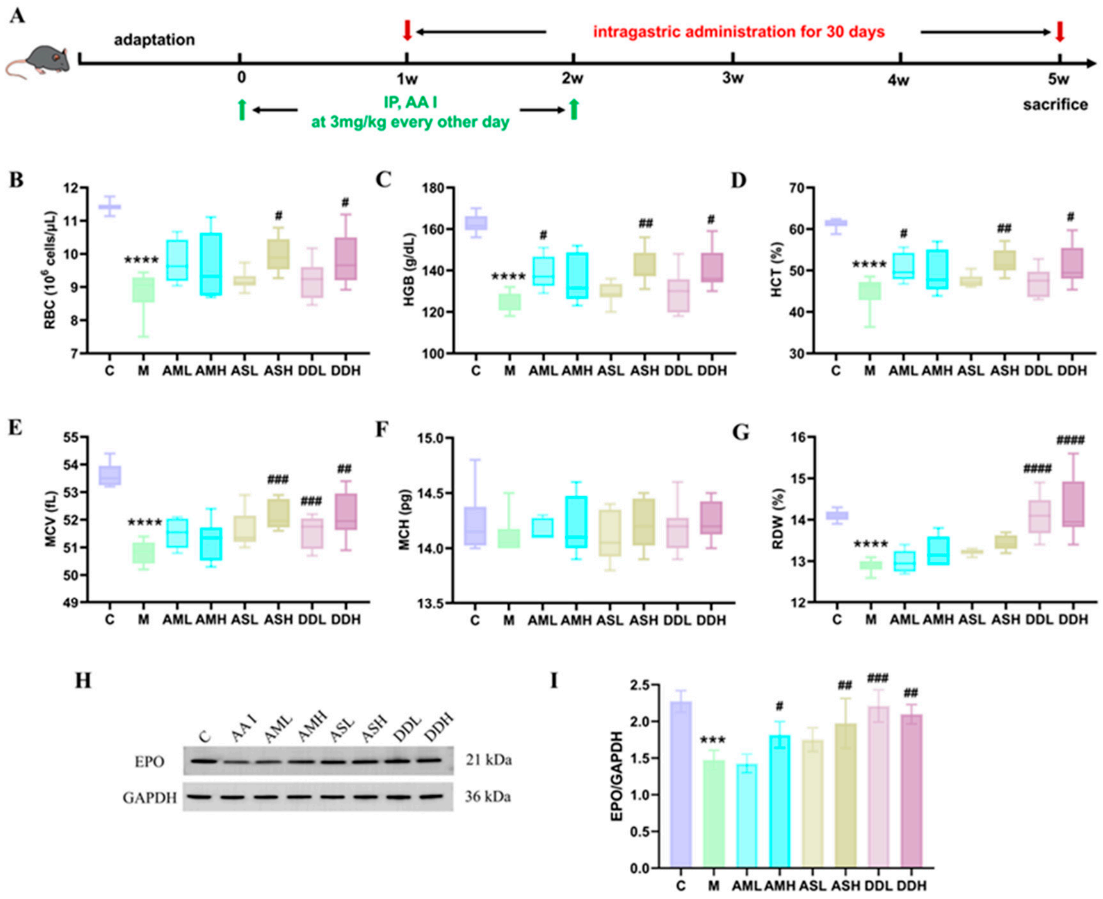

Figure S2. DD relieved AA I induced anemia. **(A)** Simplified diagram of mice administrated with test drugs. **(B-G)** Hematology of mice in control, AA I, AML, AMH, ASL, ASH, DDL, and DDH group, respectively. RBC (10^6 cells/μL), HGB (g/dL), HCT (%), MCV (fL), MCH (pg), and RDW (%). **(H-I)** The protein expression of EPO in control, AA I, AML, AMH, ASL, ASH, DDL and DDH groups, respectively. Statistical comparisons: ***, $P < 0.001$; ****, $P < 0.0001$ *vs* control. $^{\#}$, $P < 0.05$; $^{\#\#}$, $P < 0.01$; $^{\#\#\#}$, $P < 0.001$; $^{\#\#\#\#}$, $P < 0.0001$ *vs* model (n=8 for hematology and n=3 for western blotting).

The PLT and PCT in AA I group were significantly increased compared with the control group ($P < 0.01$ or $P < 0.001$). The continuous abnormal elevation of platelets may lead to an increase in blood viscosity, affecting the fluidity of blood and causing microcirculation disorders. No significant improvement was observed in PLT and PCT in treated groups. PDW was significantly decreased in AML, AMH, and ASH groups ($P < 0.05$, $P < 0.01$, $P < 0.001$). Significant increases of WBC were observed in ASL and ASH groups ($P < 0.001$) (**Figure S3**), which suggested that AS may stimulate the immune system, resulting in an increase of WBC. There was no significant difference in BASO between AA I group and the control group, whereas it decreased obviously in DDL and DDH group ($P < 0.01$). In addition, no obvious difference was observed in MCH, MONO and MPV between each group.

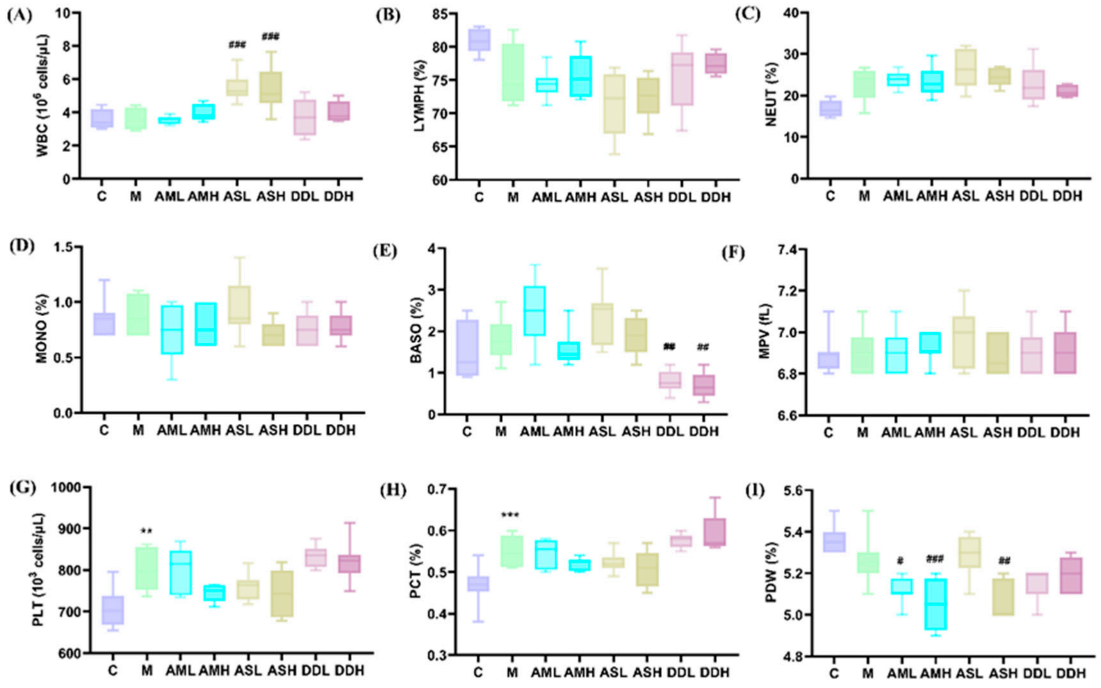

Figure S3. Hematology of mice in control, AA I, AML, AMH, ASL, ASH, DDL, and DDH group, respectively. WBC (10^6 cells/μL), LYMPH (%), NEUT (%), MONO (%), BASO (%), and MPV (fL), PLT (10^6 cells/μL), PCT (%), and PDW (%). Significantly different from the control group (**, $P < 0.01$; ***, $P < 0.001$; n=8). Significantly different from AA I group ($^{\#}$, $P < 0.05$; $^{\#\#}$, $P < 0.01$; $^{\#\#\#}$, $P < 0.001$; n=8).

3.3 Molecular docking

Table S4. Docking scores of 17 active components.

**No.**	**Compounds**	**HIF-1α（kcal/mol）**	**TGF-β (kcal/mol)**
1	quercetin	-6.01	-7.24
2	kaempferol	-5.56	-7.04
3	calycosin-7-O-glucoside	-6.67	-6.96
4	ferulic acid	-4.93	-6.43
5	formononetin	-5.17	-7.75
6	levistolide A	-5.35	-7.16
7	ononin	-6.25	-7.56
8	rutin	-6.30	-7.57
9	β-sitosterol	-5.06	-6.74
10	isorhamnetin	-5.96	-7.57
11	rhamnocitrin	-5.22	-7.30
12	vanillin	-4.25	-5.36
13	chlorogenic acid	-6.26	-6.72
14	ligustilide	-5.03	-7.26

15	vanillic acid	-4.66	-5.82
16	succinic acid	-3.83	-4.31
17	linolenic acid	-4.18	-5.80
